# Self-adaptive forward-forward network for anomaly detection and medical image analysis

**DOI:** 10.3389/fradi.2026.1771850

**Published:** 2026-06-11

**Authors:** Johanna P. Müller, Matthew Baugh, Bernhard Kainz

**Affiliations:** 1 IDEA Lab, Department of Artificial Intelligence in Biomedical Engineering, Friedrich-Alexander Universität Erlangen-Nürnberg, Erlangen, Germany; 2 Biomedical Image Analysis Group, Department of Computing, Imperial College London, London, United Kingdom

**Keywords:** anomaly detection, domain-shift detection, forward-forward learning, medical image analysis, resource-efficient neural networks, self-supervised learning

## Abstract

**Introduction:**

Robust anomaly and out-of-distribution (OOD) detection in radiology demands learning methods that are accurate, interpretable, computationally efficient, and reliable under real-world distributional shifts. Existing back-propagation-trained models often struggle to meet these requirements simultaneously, while forward-forward learning, despite its conceptual appeal as a resource-efficient and biologically plausible alternative, has so far seen limited adoption in image-based and safety-critical medical applications due to scalability and generalisation limitations.

**Methods:**

We revisit back-propagation-free learning for the open-world clinical setting and discuss the Convolutional Forward-Forward Algorithm (CFFA), a parameter-efficient reformulation of the Forward-Forward Algorithm tailored to high-dimensional medical image analysis. CFFA incorporates convolutional structure and layer-wise local objectives, overcoming key scalability and generalisation limitations of existing forward-forward approaches while retaining their resource-efficient training paradigm. Building on the observation that the forward-forward objective yields intrinsic and interpretable goodness statistics that directly quantify conformity to the learned data distribution, we introduce SaFF-AD, a self-adaptive forward-forward network explicitly designed for anomaly and OOD detection. SaFF-AD autonomously configures optimisation dynamics, architectural depth, and goodness normalisation, enabling stable learning under constrained computational budgets and in one-shot training regimes.

**Results:**

Extensive experiments across multiple medical imaging benchmarks demonstrate that SaFF-AD achieves competitive or superior anomaly detection performance compared to back-propagation-trained models, while requiring substantially fewer parameters and forward evaluations. The forward-forward goodness signal enables self-supervised anomaly and OOD detection without auxiliary networks, post-hoc uncertainty estimation, or heuristically designed scoring functions.

**Discussion:**

These results establish forward-forward learning as a viable and practically attractive alternative to conventional deep learning for safety-critical medical image analysis, particularly in settings characterised by constrained computational budgets, limited labelled data, and distributional uncertainty. By treating anomaly detection as an intrinsic property of the learned model rather than a post-hoc addition, SaFF-AD offers a unified framework that is interpretable, efficient, and well-suited to the open-world conditions encountered in real-world clinical deployment.

## 1 Introduction

Global healthcare disparities demand machine learning systems that are accurate, computationally efficient, and robust to the distributional variability encountered in real-world clinical practice. In radiology, imaging protocols, scanner hardware, patient populations, and disease prevalence vary substantially across institutions, while access to centralised compute infrastructure remains uneven. As a result, many state-of-the-art diagnostic models struggle to generalise beyond their training environments and remain difficult to deploy in settings with constrained energy budgets, limited hardware, or systematically shifted data distributions (1–4).

A key challenge underlying this gap is the open-world nature of clinical imaging. In routine practice, models are frequently confronted with inputs that do not conform to their training distribution, including rare diseases, incidental findings, demographic shifts, and scanner-specific artefacts. In this setting, anomaly detection has emerged as an important capability. Rather than directly assigning a predefined diagnostic label, anomaly detection asks a more fundamental question: does an image conform to what the model has learned as normal? This formulation is particularly well suited to medical imaging, where clinically relevant abnormalities are rare, heterogeneous, and often not fully defined at training time. Unlike standard classification, which assumes a closed and well-specified set of disease categories, anomaly detection must operate under limited supervision and substantial uncertainty about what constitutes abnormality (5–13).

We therefore view anomaly detection as a self-supervised out-of-distribution detection (OOD) problem that naturally precedes anomaly or pathology categorisation (8, 11, 14). By learning a representation of the normative data distribution from predominantly healthy or unlabelled data, a model can identify statistically meaningful deviations at test time without requiring explicit labels for every possible pathology. This separation between detecting something that is unusual and determining what it is mirrors clinical reasoning and provides a robust first line of defence against unseen diseases, rare conditions, and domain shifts. For deployment in radiology, however, such systems must additionally be interpretable, computationally tractable, and operable under strict resource constraints (7, 9, 15).

In this context, we revisit back-propagation-free learning through the Forward-Forward Algorithm (FFA) (16). FFA replaces global error back-propagation with layer-wise local objectives, offering a biologically inspired and resource-efficient alternative to conventional training. Despite its conceptual appeal, existing forward-forward approaches have seen limited adoption in image-based and safety-critical domains due to scalability challenges and reduced generalisation performance.

We show that these limitations are not intrinsic to forward-forward learning, but rather arise from architectural and objective-level design choices. We discuss the *Convolutional Forward-Forward Algorithm (CFFA)*, which incorporates a convolutional structure to enable scalable representation learning for high-dimensional medical images and we demonstrate that the forward-forward goodness signal is not only a training construct, but constitutes an intrinsic and interpretable measure of conformity to the learned data distribution.

Building on this insight, we introduce *SaFF-AD*, a self-adaptive forward-forward network explicitly designed for self-supervised anomaly and out-of-distribution (OOD) detection in medical imaging, where anomaly detection asks whether an input deviates from the learned normative distribution of healthy data, and OOD detection refers to the broader mechanism of identifying inputs that lie outside the training distribution, including domain shifts, scanner artefacts, and unseen pathologies that may not constitute a clinical anomaly per se. We treat anomaly detection as a self-supervised OOD problem: the model is trained exclusively on normal (healthy) images, using Poisson Image Interpolation [PII, (17)] to generate synthetic negative samples. PII is a well-established strategy in medical anomaly detection (7, 17, 18) that produces diverse, non-deterministic local irregularities not tied to specific disease patterns, enabling generalisation to unseen anomaly types at test time. SaFF-AD autonomously configures optimisation dynamics, architectural depth, and goodness normalisation based on data characteristics and resource constraints, enabling stable learning in low-compute and one-shot regimes while improving robustness to distributional shifts.

We substantially extend our earlier work on self-adapting Forward-Forward Networks, SaFF-Net (15), which focused on architectural efficiency, classification, and pretraining under constrained computational budgets. To the best of our knowledge, anomaly detection has not been treated as a primary learning objective in forward-forward learning so far. In this work, anomaly and OOD detection are repositioned as the central task, accompanied by a dedicated self-supervised anomaly objective, extended baseline comparisons, and a substantially expanded experimental evaluation spanning classification, pretraining, and anomaly detection across multiple benchmarks. Our contributions are summarised as follows:

1. We formulate anomaly and OOD detection as an intrinsic, self-supervised consequence of forward-forward learning, rather than as a post-hoc uncertainty or scoring mechanism, and define their relationship explicitly: OOD detection is the general mechanism; anomaly detection is its clinically motivated instantiation.
2. We discuss CFFA, extending the Forward-Forward Algorithm of (16) to scalable image-based architectures suitable for medical imaging, and evaluate on classification and pretraining tasks.
3. We demonstrate that the forward-forward goodness signal provides an interpretable and resource-efficient anomaly score, enabling reliable detection of unseen abnormalities and domain shifts on the AADD benchmark (13).
4. We propose *SaFF-AD*,^1^ a self-adaptive forward-forward network for anomaly and OOD detection, and evaluate its robustness, computational efficiency, and detection performance, alongside classification and pretraining, on multiple medical imaging benchmarks.

### 1.1 Related work

Recent work has extended FFA beyond its original formulation to more practical and scalable architectures. In particular, various approaches in literature have demonstrated that convolutional neural networks can be trained with forward-forward learning by incorporating spatially structured objectives and extended label representations, achieving competitive performance on standard image benchmarks such as MNIST, CIFAR-10, and CIFAR-100 (19–21).

Complementary efforts have focused on improving computational efficiency and hardware compatibility. Forward Learning of Graph Neural Networks (ForwardGNN) shows that FF-style layer-wise local objectives can be successfully applied to graph-structured data, substantially broadening the scope of forward-forward learning beyond image-based models (22). Extensions to spiking neural networks and distance-metric-based formulations further enhance biological plausibility and enable energy-efficient, low-power implementations, making forward-forward learning attractive for resource-constrained environments (23, 24). Additionally, the Self-Contrastive Forward-Forward algorithm integrates contrastive and self-supervised learning principles by constructing positive and negative examples directly from raw data, enabling representation learning without explicit labels (25). These developments indicate a growing interest in forward-forward learning as a general-purpose paradigm that extends beyond supervised classification and into open-world and self-supervised settings.

Out-of-distribution (OOD) detection methods are commonly built around models trained on in-distribution medical imaging data, but are rarely purely supervised; instead, they combine supervised objectives with complementary strategies such as feature-space distance measures [e.g., Mahalanobis-based scoring in (26)], uncertainty-aware or label-efficient modelling (27), and information-theoretic or representation-based criteria for detecting distributional shifts (28), as well as broader hybrid paradigms surveyed in (29). These approaches exploit intermediate feature representations, predictive uncertainty, or auxiliary training signals to improve robustness to unseen samples while reducing reliance on fully labelled datasets. In parallel, self-supervised learning (SSL) has become a widely adopted strategy for anomaly detection, especially in medical imaging, where labelled anomalies are scarce. Methods such as CutPaste by (30) and Poisson Image Interpolation by (17) create synthetic outliers to train models to detect deviations from normal patterns. Natural Synthetic Anomalies extend this approach by generating realistic synthetic anomalies, enabling robust representation learning for both detection and localisation (18). Multi-task synthetic frameworks further improve generalisation across anomaly types (7), while confidence-aware SSL techniques integrate uncertainty estimation for safer anomaly detection in clinical settings (11). Benchmarking frameworks such as nnOOD (8), MedIAnomaly (10), and NOVA (9) provide standardised evaluation protocols for SSL-based anomaly localisation, supporting reproducibility and fair comparison.

Generative and reconstruction-based approaches have established a foundational paradigm for unsupervised OOD detection (31–35). These methods typically learn the manifold of healthy anatomical variability, identifying anomalies as deviations from this learned norm. Early seminal work like f-AnoGAN employs Generative Adversarial Networks to model normal image appearance, flagging pathologies through high reconstruction and discriminator residual errors (36). Similarly, normative modelling using Variational Auto-Encoders has been proposed to localise anomalies by analysing pixel-wise Kullback-Leibler divergence (37), while spatial autoencoders have demonstrated the ability to capture complex anatomical regularities across diverse pathologies without disease-specific labels (38). Recent advances emphasise that OOD detection performance is highly sensitive to the choice of reconstruction metric; for instance, ensembles utilising structural similarity (SSIM) and perceptual metrics (LPIPS) have proven significantly more effective at identifying subtle artefacts in brain MRI than standard pixel-wise losses (39, 40) show that pixel-wise $l^{\;p}$-difference fails to detect anomalies that are not characterised by large intensity differences to the surrounding tissue.

## 2 Materials and methods

### 2.1 Forward-forward algorithm

**Figure 1:**
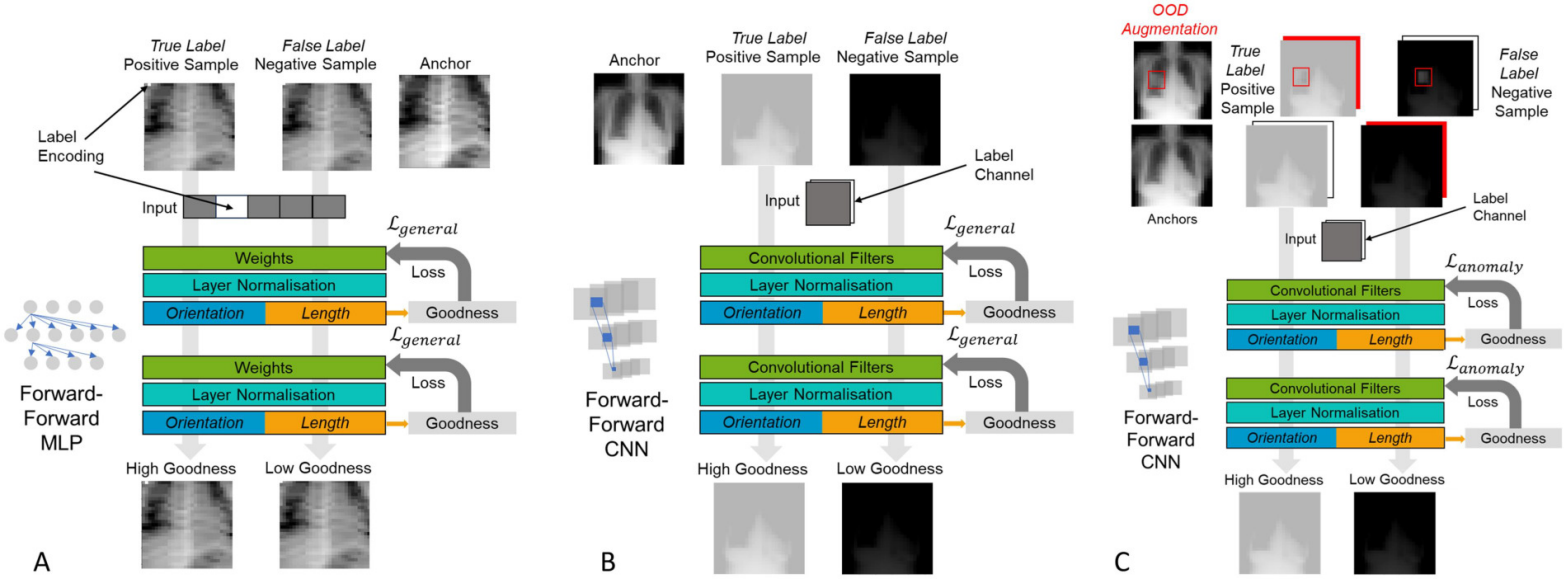
Forward-Forward architectures and training variants included in SaFF-AD. **(A)** Forward-Forward multi-layer perceptron (FF-MLP). **(B)** Forward-Forward convolutional neural network (FF-CNN) (15). **(C)** FF-CNN trained with an anomaly loss using a synthetic task in which anomalous versions of the anchor input are generated. All models are trained layer-wise. An anchor image (before label encoding and augmentation) is used to produce positive and negative samples via label encoding and corresponding augmentations. Each layer applies a type of normalisation, separating activations into orientation, which is forwarded to the next layer, and magnitude, which defines the layer goodness. Layers are optimised to yield high goodness (above a threshold) for positive samples and low goodness (below a threshold) for negative samples. During inference, classification is performed by summing goodness scores across all layers except the first, evaluated for each candidate label.

FFA by (16) (Figure 1, left) is a multi-layer, back-propagation-free learning paradigm inspired by Boltzmann machines (41) and Noise Contrastive Estimation (42). Unlike traditional backpropagation, FFA uses two forward passes with opposing objectives: a positive pass on real (true) data $x^{+}$ to increase the *goodness* and a negative pass on counter-examples $x^{-}$ to decrease it. The goodness function (Equation 1) for a hidden layer is defined as

$$g(x)=\sum_{\;j}y_{j}(x^{+/-}{)}^{2}-\theta ,$$ (1)

where $y_{j}$ is the activation of hidden unit j and θ is a learnable threshold. The probability that an input x is real (true) is given by p(x)=σ(g(x)), where σ is the logistic function. To prevent trivial separability between positive and negative passes when propagating activations to subsequent layers, the FFA normalises the length of the hidden vector, forwarding only the orientation while using the vector length for goodness evaluation. This layer-wise local objective enables efficient, biologically plausible learning, and facilitates learning on resource-constrained devices.

### 2.2 Convolutional forward-forward algorithm

We extend FFA to convolutional architectures with as CFFA, preserving spatial structure in feature maps (Figure 1, right). For a 2D convolutional layer (Equation 2):

$$y_{i,j}=\alpha \left(\sum_{m=1}^{M}\sum_{n=1}^{N}x_{m,n}\cdot w_{i,j,m,n}+b_{i,j}\right),$$ (2)

where $y_{i,j}$ is the output feature at position (i,j), $w_{i,j,m,n}$ the kernel weight, $b_{i,j}$ the bias, and α(⋅) the activation. Goodness is computed from the vector length $y_{i,j}^{+/-}$, and orientation is normalised before forwarding to the next layer, maintaining spatial dependencies. CFFA naturally supports efficient convolutional feature extraction and is compatible with on-chip, low-power training for edge devices (43, 44). This allows scalable, high-dimensional image analysis without the overhead of backpropagation.

### 2.3 Objective function

We optimise a *goodness* function $g_{\theta }(\cdot )$ for each hidden layer. For general applications such as classification or contrastive pretraining, the network is trained to increase the goodness for positive samples $x^{+}$ and decrease it for negative samples $x^{-}$. We adopt a threshold-based formulation (Equation 3):

$$L_{\text{general}}=\frac{1}{B}\sum_{i=1}^{B}\log\left(1+\exp\left([-g_{\theta }(x_{i}^{+})+\tau ,\;g_{\theta }(x_{i}^{-})-\tau ]\right)\right),$$ (3)

where B is the batch size, τ is a trainable threshold parameter, and the concatenation inside the exponential penalises positive examples whose goodness falls below the threshold and negative examples whose goodness exceeds it. This formulation generalises standard contrastive or cross-entropy objectives while remaining fully local and back-propagation-free.

### 2.4 Self-supervised anomaly detection

Let $D_{\text{normal}}=\{x_{i}{\}}_{i=1}^{N}$ denote a dataset of normal (non-anomalous) medical images. In self-supervised anomaly detection, the model is trained exclusively on $D_{\text{normal}}$ to learn a representation of the normal data distribution. To enable the detection of abnormal patterns without relying on labelled anomalies, we introduce synthetic anomalous samples generated via domain-relevant augmentation for mimicking aberrations of the norm, like lesions.

Formally, for each normal sample $x\in D_{\text{normal}}$, we construct a synthetic anomaly $x^{anomalous}=T(x)$, where T(⋅) is an augmentation designed to mimic realistic anomalies. Examples include Poisson image interpolation (17), patch swapping or texture perturbations (18), and multi-task synthetic variations (7). The resulting self-supervised training set is

$$D_{\text{SSL}}=\{(x^{+},x^{-})\;:\;x^{+}\in D_{\text{normal}},x^{-}=T(x^{+})\}.$$

As for classification tasks, positive samples $x^{+}$ are correctly and $x^{-}$ incorrectly, or oppositely for anomaly detection, labelled samples.

Given a forward-forward goodness function $g_{\theta }(\cdot )$ parameterised by θ, we optimise the network to increase the goodness for positive examples $x^{+}$ while decreasing it for negative samples $x^{-}$. In contrast to standard contrastive objectives, we employ the inverse threshold-based FFA loss (Equation 4):

$$L_{\text{anomaly}}=\frac{1}{\left|D_{\text{SSL}}\right|}\sum_{(x^{+},x^{-})\in D_{\text{SSL}}}\log\left(1+\exp\left([g_{\theta }(x^{+})-\tau ,\;\tau -g_{\theta }(x^{-})]\right)\right),$$ (4)

where τ is a trainable threshold, and the concatenation inside the exponential penalises positive examples whose goodness falls below τ and synthetic negatives whose goodness exceeds τ. The trainable parameter τ reduces the need for manually defined goodness thresholds and encourages the network to learn a robust separation between normal and anomalous patterns.

At test time, unseen images $x_{\text{test}}$ are scored using the learned goodness $g_{\theta }(x_{\text{test}})$, with lower values indicating a higher likelihood of being anomalous. This approach naturally integrates self-supervised anomaly generation into the forward-forward learning paradigm, enabling detection and localisation of out-of-distribution patterns in radiological imaging without reliance on labelled anomalies.

### 2.5 Self-adaptive forward-forward network for anomaly detection

We define *SaFF-AD* as a self-adaptive forward-forward network that jointly optimises representation learning, architectural configuration, and anomaly discrimination under resource constraints, alongside other applications like classification and pretraining. Let $f_{\theta }=f_{\theta_{L}}\circ \cdots \circ f_{\theta_{1}}$ denote a forward-forward network composed of L convolutional blocks, each parameterised by $\theta_{\ell }$ and associated with a local goodness function $g_{\theta_{\ell }}(\cdot )$. SaFF-AD instantiates forward-forward learning using convolutional operators and integrates the self-supervised anomaly objective into all stages of optimisation and inference.

Rather than fixing the network depth L a priori, SaFF-AD treats L as a data-dependent variable. During training, candidate depths $L\in \{1,\ldots ,L_{\max}\}$ are evaluated incrementally. For each depth L, optimisation proceeds by minimising the self-supervised anomaly loss defined in Equation 3 using layer-wise updates. Let $\Delta_{L}$ denote a goodness separation criterion measured on a validation set, for example, the difference between expected goodness values for normal and synthetic anomalous samples,

$$\Delta_{L}=E_{x^{+}}[g_{\theta }(x^{+})]-E_{x^{-}}[g_{\theta }(x^{-})].$$

Training is terminated early at depth L if improvements in $\Delta_{L}$ fall below a predefined tolerance, or if validation stability degrades. This hierarchical early stopping mechanism is applied at the layer, epoch, and depth levels, yielding an adaptive trade-off between representational capacity and computational cost.

Optimisation dynamics are stabilised through adaptive normalisation of goodness and threshold learning. In particular, the trainable threshold τ introduced in the anomaly objective is jointly optimised with the network parameters θ, resulting in a coupled optimisation problem (Equation 5)

$$\min_{\theta ,\tau }\;L_{\text{anomaly}}(\theta ,\tau ).$$ (5)

This coupling aligns training and inference objectives and removes the need for manually selected decision thresholds. To ensure numerical stability and consistent activation statistics, Peer-Normalisation and Batch-Normalisation terms are incorporated directly into the forward-forward objective at each layer, constraining the distribution of intermediate representations during training.

SaFF-AD further incorporates structured network pruning (45) as part of its adaptive optimisation strategy. Let $\theta_{\ell }$ denote the parameters of block ℓ. Pruning decisions are guided by the marginal contribution of each block to the goodness separation criterion, quantified as $\delta_{\ell }=\Delta_{L}-\Delta_{L\setminus \ell },$ where $\Delta_{L\setminus \ell }$ denotes the separation achieved when block ℓ is removed or masked. Blocks with $\delta_{\ell }$ below a predefined threshold are pruned, reducing parameter count and inference cost without degrading anomaly detection performance.

During an initial warm-up phase, multiple candidate configurations (L,θ,τ) are evaluated using statistically grounded selection criteria based on validation goodness separation, convergence behaviour, and stability across batches. The configuration that maximises goodness separation subject to computational constraints is selected for full training.

At inference time, an unseen image $x_{\text{test}}$ is assigned an anomaly score given directly by the learned goodness function $g_{\theta }(x_{\text{test}})$. Samples with low goodness values are interpreted as deviations from the learned normative distribution. By jointly optimising representation learning, architectural configuration, and anomaly discrimination within the forward-forward paradigm, SaFF-AD yields a unified, resource-efficient framework in which anomaly and out-of-distribution detection emerge as intrinsic properties of the learned model rather than as post-hoc additions.

### 2.6 Datasets

**Figure 2:**
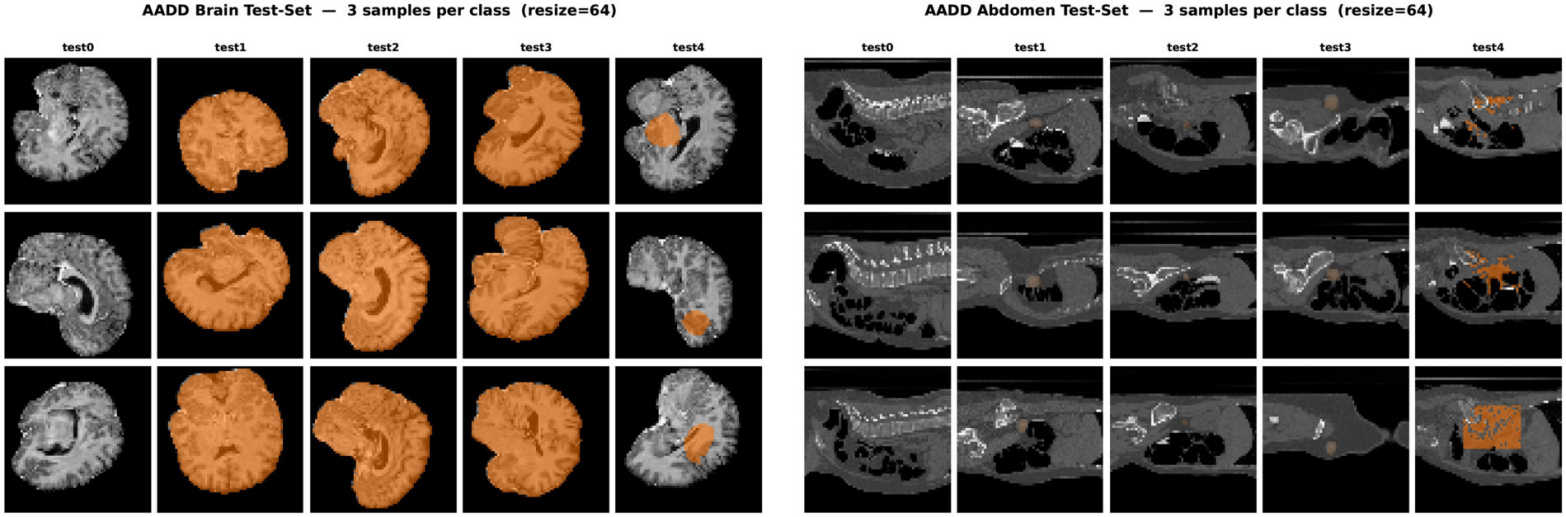
Anomaly location in the AADD Dataset, in orange. **(Left)** Brain dataset. Normal samples have a band of spatial frequencies removed and are rotated about a random axis by an angle in the range of [30,60] degrees (*test0*). Anomalous samples are over-rotated by an angle in the range of [75,105] degrees (*test1*); under-rotated by an angle in the range of [0,15] degrees (*test2*); or rotated normally but including additional spatial frequencies (*test3*); or with spatial frequencies added only within a certain region (*test4*). **(Right)** Abdomen dataset. Normal samples include a new synthetic organ (*test0*). Anomalous samples include added texture to the synthetic organ (*test1*); split morphology into two smaller organs (*test2*); normal organ in an abnormal location (*test3*); and the complete absence of the organ (*test4*) (13, 37, 49, 50).

We evaluate *SaFF-AD* using a combination of standard benchmarks and medical imaging datasets, covering both classification and anomaly detection tasks. *MNIST* (46) serves as a baseline for validating training stability and convergence on simple, low-dimensional images. *MedMNIST* (47) provides a variety of preprocessed medical imaging subsets, enabling evaluation on binary classification tasks (healthy vs. unhealthy) and small-scale segmentation tasks. For high-resolution clinical radiographs, we employ *VinDr-CXR* (48), which contains 100,000 thoracic chest X-ray images annotated by 17 radiologists; we focus on binary classification (healthy vs. unhealthy) and Cardiomegaly segmentation. To specifically benchmark self-supervised anomaly detection, we include the *Advancing Anomaly Detection Dataset (AADD)* (13) based on original data by (37, 49, 50). *AADD* contains volumetric brain MRI and abdominal CT scans, with training data restricted to normal images and testing data including multiple types of synthetic anomalies, see Figure 2. For anomaly detection on *AADD*, only normal images from the training split are used, while testing is conducted on both normal and anomalous subsets (“test0” for normal, “test1-test4” for anomalies). For brain, normal samples have a band of spatial frequencies removed and are rotated about a random axis by an angle in the range of [30,60] degree (*test0*). Anomalous samples are over-rotated by an angle in the range of [75,105] degrees (*test1*); under-rotated by an angle in the range of [0,15] degrees (*test2*); or rotated normally but including additional spatial frequencies (*test3*); or with spatial frequencies added only within a certain region (*test4*). For abdomen, normal samples include a new synthetic organ (*test0*). Anomalous samples include added texture to the synthetic organ (*test1*); split morphology into two smaller organs (*test2*); normal organ in an abnormal location (*test3*); and the complete absence of the organ (*test4*).

### 2.7 Pre-processing

**Figure 3:**
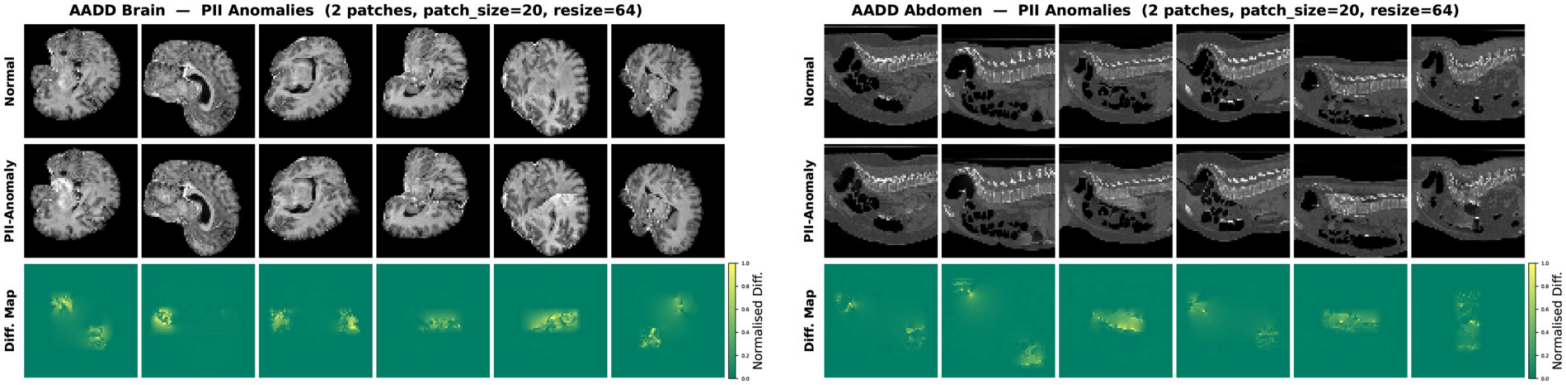
Anomalies genherated by Poisson Image Interpolation (PII) on the AADD Dataset. With PII, we simulate anomalies in an open-world setup through perturbations of the normal images with smooth blending (17). **(Left)** Brain dataset. **(Right)** Abdomen dataset.

All inputs are Z-score normalised using per-dataset mean and standard deviation statistics, either provided as fixed constants or estimated automatically by computing the mean and standard deviation of a single training mini-batch at the start of each run. Where established values exist, these are used: MNIST images are normalised with μ=0.1307, σ=0.3081, and AADD images with μ=0.13, σ=0.14; all other datasets rely on automatic estimation. Two-dimensional images are resized to 28×28 (MedMNIST, MNIST) or 256×256 (VinDr-CXR) using bilinear interpolation, with VinDr-CXR DICOM files first decoded via their embedded VOI look-up table and subsequently min–max scaled to [0,1]. Volumetric inputs are resized to a configurable resolution (e.g., $64^{3}$–$128^{3}$ voxels) using trilinear interpolation and then normalised per volume as $\hat{v}=(v-\mu_{v})/(\sigma_{v}+\varepsilon )$, where $\varepsilon =10^{-8}$, in order to mitigate scanner-level intensity offsets. The Forward–Forward algorithm requires explicit positive and negative data pairs. Under full supervision, a one-hot class label is embedded directly into the input, and negative samples are formed by permuting images across the mini-batch whilst keeping each sample’s original label, making the label–image pairing deliberately incorrect. In the unsupervised setting, negatives are anomalous images produced by blending each sample with a permuted counterpart using a spatially smooth binary mask obtained through iterative Gaussian filtering and thresholding. For self-supervised anomaly detection, pseudo-anomalous samples are generated by Poisson blending patches from a permuted image into the original, see Figure 3, covering 20% of all patches by default. We apply mild transformation through random rotation by [−10,10] degrees.

### 2.8 Baselines

Baseline architectures are detailed in the Supplementary Material. Briefly, the MLP baseline comprises 2,797,010 parameters, the CNN baseline 1,554,954 parameters, and ResNet-18 11,183,694 parameters, serving as representative points of comparison across parameter scales. All three baselines are trained end-to-end via standard backpropagation using the Adam optimiser with cross-entropy loss and were not pretrained. Training follows the same batch regime as for our method, with early stopping, and no data augmentation besides random rotation by 10 degrees is applied, ensuring comparability with the proposed method. These baselines collectively represent standard supervised learning under backpropagation and provide reference accuracy values against which the Forward-Forward-based models are evaluated.

For the self-supervised anomaly detection task on the AADD dataset, the same backbone architectures are repurposed as feature extractors and trained using synthetically generated out-of-distribution (OOD) samples as the only supervisory signal. Pseudo-anomalies are created by blending image patches from different training samples into each input via Poisson image editing (17), yielding seamless yet semantically inconsistent composites (PII synthesis, patch ratio r=0.35, patch size 8×8). The choice of training regime is motivated by the desire to disentangle two orthogonal design axes: how the model is trained and how anomaly scores are derived at test time. Binary cross-entropy provides the most direct discriminative baseline, framing the problem as classification between real and synthetically corrupted samples, with the anomaly score given by the softmax probability assigned to the anomaly class. The EBM regime is motivated by the theoretical appeal of energy-based formulations (51), which explicitly encourage compact energy for normal data and high energy for OOD inputs without requiring softmax normalisation over all classes; the feature extractor is trained with (Equation 6)

$$L_{\text{EBM}}={\left[E(f_{\text{in}})-m_{\text{in}}\right]}_{+}+{\left[m_{\text{out}}-E(f_{\text{out}})\right]}_{+}+\lambda \left(E(f_{\text{in}}{)}^{2}+E(f_{\text{out}}{)}^{2}\right),$$ (6)

where $E(f)=|f{|}^{2}/D$ is the mean squared feature norm, $m_{\text{in}}=5$, $m_{\text{out}}=20$, and λ=0.01. Two scoring strategies are evaluated on top of this shared backbone: in the *EBM* variant the anomaly score is the energy value E(f) directly, exploiting the fact that the loss explicitly pushes in-distribution features towards low energy; in the *EBM+Mahalanobis* variant the energy head is discarded at test time and replaced by the Mahalanobis distance (52) in feature space, with covariance estimated via Ledoit-Wolf shrinkage (53) on the training-set features. The *CE+Mahalanobis* variant follows the same cross-entropy training but discards the classification head at test time, replacing the softmax score with the Mahalanobis distance in the penultimate feature space; this configuration is included because discriminatively trained representations have been shown to yield well-structured feature spaces in which density-based scoring outperforms the corresponding softmax probabilities (52) computed in the final D=256-dimensional projected feature space (the output of the shared projection head), and pairing it with the *CE* backbone tests whether this benefit transfers to the self-supervised PII setting. Together, these four regimes constitute the self-supervised backpropagation baselines against which the proposed Forward-Forward-based models are evaluated.

### 2.9 Implementation details

**Figure 4:**
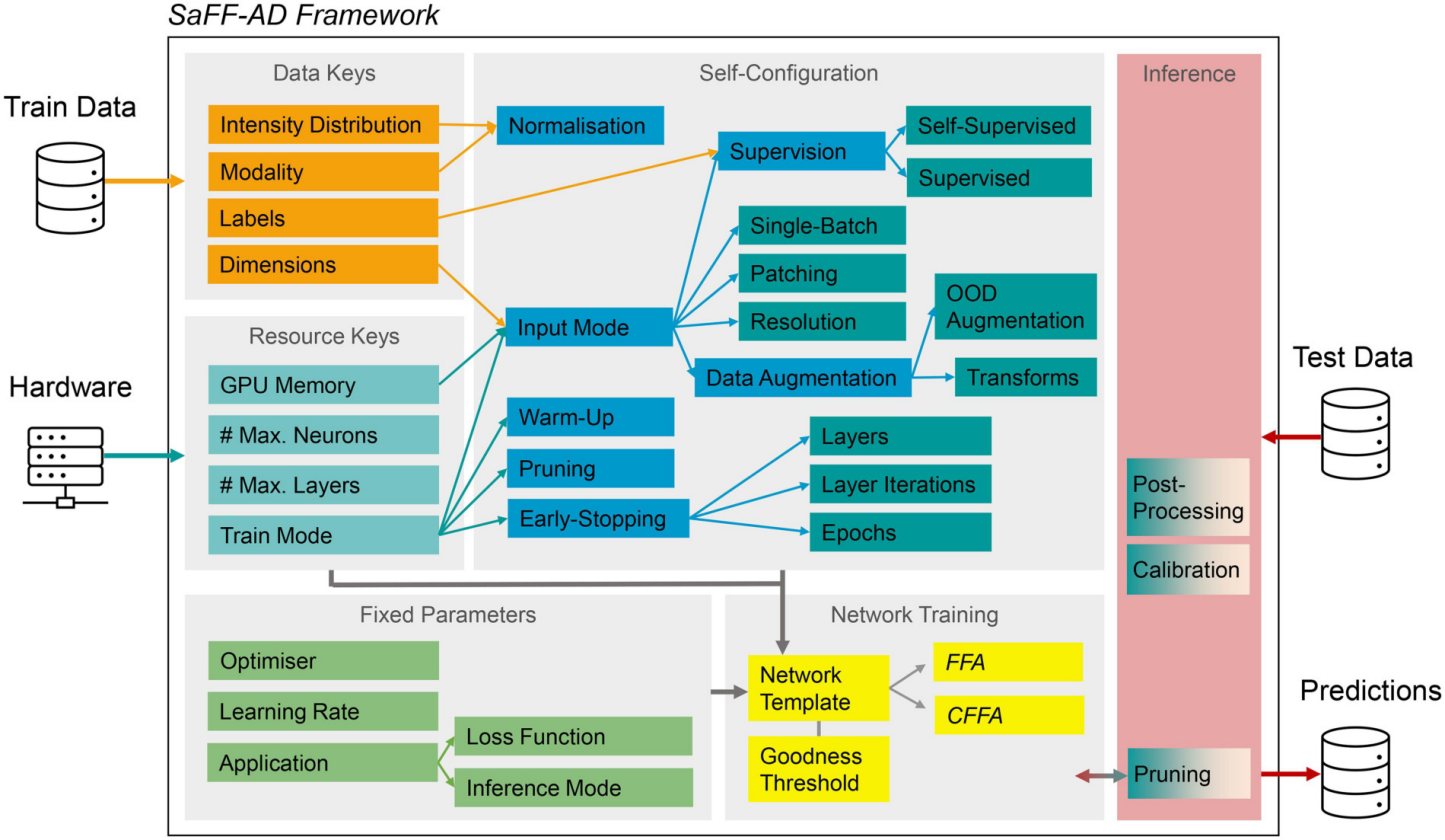
SaFF-AD Framework. End-to-end pipeline for efficient anomaly detection and representation learning, such as classification and pretraining. Data characteristics and choice of application guide the self-configuration of resource-aware model parameters. Training supports supervised and self-supervised modes with patching, normalisation, pruning, and early stopping for 1D, 2D, and 3D FFA/CFFA architectures; the goodness threshold is trainable. Inference produces anomaly scores with optional post-processing, enabling deployment across heterogeneous and resource-constrained systems.

Figure 4 provides an overview of the end-to-end SaFF-AD pipeline, illustrating how the self-supervised anomaly detection objective is embedded within the self-adaptive forward-forward training and inference framework. This unified framework also supports additional applications, including classification and representation pretraining.

At training time, dataset characteristics such as modality, dimensionality, spatial resolution, and intensity distribution are first analysed and used to initialise a resource-aware configuration. This self-configuration step determines architectural and optimisation parameters, including network depth, number of convolutional forward-forward blocks, patching strategy, and memory allocation. As shown in Figure 4, these decisions directly constrain GPU memory usage and batch size, enabling stable training across 1D, 2D, and 3D imaging settings using the same underlying framework.

To further improve efficiency and stability, SaFF-AD integrates structured network pruning, Peer-Normalisation, and Batch-Normalisation directly into the optimisation objective. These components regularise layer-wise representations, stabilise goodness statistics across batches, and reduce parameter count without degrading anomaly detection performance. The resulting architecture is therefore jointly optimised for anomaly discrimination, computational efficiency, and deployment robustness.

All experiments are implemented in Python using PyTorch and executed on NVIDIA A100 GPUs with 80 GB of memory. Training time depends on dataset size, network depth, and the number of layer-wise optimisation iterations. One-shot learning scenarios typically complete in under 30 min per model, while full training runs complete within approximately two hours. Batch sizes are selected dynamically based on input resolution and memory constraints. By integrating self-configuration, adaptive optimisation, and forward-forward learning within a unified pipeline, SaFF-AD not only enables practical and scalable anomaly and OOD detection but also classification and representation pretraining across diverse radiological imaging scenarios.

## 3 Results

### 3.1 Ablation study

#### 3.1.1 FFA backbone

Across both datasets, see Table 1, SaFF-AD consistently improves accuracy and AUC over the FFA baseline, with gains of up to several hundred percent in the one-shot MNIST setting and modest single-digit percentage improvements in standard training. Early stopping on iterations yields the largest computational benefit, reducing the number of function evaluations by over 80%–90% while maintaining or improving performance in one-shot training, but causes substantial performance degradation (over 80% drop in accuracy) in standard MNIST training. Early stopping on layers provides small accuracy improvements (≈ 1%–2%) and reduces computation by around 25%. Pruning leads to moderate accuracy drops (≈ 5%–10%) without computational savings, and retraining does not recover this loss. Warm-up significantly worsens performance (over 90% accuracy drop on MNIST) while increasing computation. Training thresholds and peer normalisation improve accuracy by a few percent and help SaFF-AD reach its best overall trade-off, achieving comparable or better performance than the baseline with roughly 60%–80% fewer function evaluations.

**Table 1:** Ablation study for *SaFF-AD* self-configuration on MNIST and MedMNIST (Supervised).

Ablation	MNIST						MedMNIST Pneumonia					
	FFA (*One-shot*)			FFA			FFA (*One-shot*)			FFA		
	ACC↑	AUC↑	NFE↓	ACC↑	AUC↑	NFE↓	ACC↑	AUC↑	NFE↓	ACC↑	AUC↑	NFE↓
Baseline	0.24	0.83	8.0	0.95	0.99	80.0	0.84	0.84	8.0	0.87	0.90	80.0
ES Iterations	0.93	0.99	**7.3**	0.11	0.55	**9.3**	**0.87**	**0.91**	**1.5**	0.83	0.89	**2.0**
ES Layers	0.24	0.83	8.0	0.96	**1.00**	60.0	0.84	0.85	8.0	0.87	0.90	60.0
ES Epoch	–	–	–	0.95	**1.00**	80.0	–	–	–	0.84	0.89	56.0
Pruning (0.4)	0.22	0.82	8.0	0.85	0.99	80.0	0.85	0.84	8.0	0.82	0.83	80.0
w/ Retraining	0.22	0.82	8.8	0.85	0.99	80.8	0.85	0.84	8.8	0.81	0.83	80.8
Warm-Up	0.01	0.42	32.0	0.64	0.93	104.0	0.80	0.86	32.0	0.83	0.91	80.0
Train. Thresh.	0.84	0.97	8.0	**0.97**	**1.00**	80.0	0.83	0.72	8.0	**0.89**	**0.94**	80.0
Peer-Norm	0.23	0.83	8.0	0.95	**1.00**	80.0	0.83	0.86	8.0	0.85	0.89	80.0
*SaFF-AD*	**0.97**	**1.00**	29.9	**0.97**	**1.00**	29.9	**0.87**	**0.91**	15.8	0.87	0.91	15.7
#Par.	1,144,004			893,503			3,788,004			2,787,003		

The FFA Baseline consists of 4 layers with 1,000 neurons each. *One-shot* means the network is trained on one single batch. Networks are trained for a maximum of 10 epochs with 1,000 layer-iterations. The optimised number of parameters for *SaFF-AD* is given in the last row. Number of function evaluations (NFE)—Forward and Backward passes $\times 1e^{3}$ during training.

ES, Early Stopping.

**1st-ranked**, 2nd-ranked.

#### 3.1.2 CFFA backbone

For CFFA, most ablations result in small performance changes, see Table 2. Early stopping on iterations reduces computation by about 20%–80%, but leads to accuracy drops of up to ∼7%–10% on MNIST and MedMNIST. Early stopping on layers reduces computation by roughly 15%–55% with minor accuracy losses (≈2%–5%). Pruning causes consistent performance degradation (≈3%–12% accuracy loss), particularly severe on MedMNIST when combined with retraining. Training thresholds slightly improve accuracy (≈2%–4%) and yield the strongest overall results on MedMNIST. Batch normalisation has minimal impact. Overall, SaFF-AD provides stable accuracy improvements of a few percent over the baseline while reducing computation, demonstrating a favourable accuracy-efficiency trade-off.

**Table 2:** Ablation Study for CFFA on MNIST and MedMNIST (Supervised).

Method	MNIST						MedMNIST Pneumonia					
	CFFA (*One-shot*)			CFFA			CFFA (*One-shot*)			CFFA		
	ACC↑	AUC↑	NFE↓	ACC↑	AUC↑	NFE↓	ACC↑	AUC↑	NFE↓	ACC↑	AUC↑	NFE↓
Baseline	**0.94**	**0.99**	80.0	0.86	0.98	80.0	0.86	0.90	80.0	0.85	0.90	80.0
ES Iterations	0.87	**0.99**	**65.2**	0.86	0.98	80.0	**0.87**	0.92	**20.0**	0.83	0.88	**14.1**
ES Layers	**0.94**	**0.99**	80.0	0.87	0.96	**66.0**	0.86	0.90	80.0	0.82	0.86	35.0
ES Epoch	–	–	–	0.86	0.98	80.0	–	–	–	0.85	0.89	80.0
Pruning (0.05)	0.91	**0.99**	80.0	0.85	**0.99**	80.0	0.84	0.89	80.0	0.85	0.90	80.0
w/ Retraining	0.93	**0.99**	80.8	0.85	**0.99**	80.8	0.76	0.81	80.4	0.85	0.90	80.8
Train. Thresh.	0.92	**0.99**	80.0	0.89	**0.99**	80.0	**0.87**	0.91	80.0	**0.87**	**0.91**	80.0
Batch-Norm	0.87	0.98	80.0	0.86	0.98	80.0	0.85	0.89	80.0	0.86	0.89	80.0
*SaFF-AD*	0.90	**0.99**	71.8	**0.90**	**0.99**	80.0	**0.87**	**0.93**	37.9	**0.87**	**0.91**	80.8

The CFFA network consists of 4 layers with [32,32,128,128] channels each (194,432 # Params). Unless otherwise noted, networks are trained max. 10 epochs with 10,000 layer iterations for CFFA(*One-shot*) and 1,000 layer iterations for CFFA. NFE—Forward and Backward passes $\times 1e^{3}$ during training.

ES, Early Stopping.

**1st-ranked**, 2nd-ranked.

#### 3.1.3 One-shot training

A practically relevant setting explored in this work is training on a single batch drawn from the full training set, without iterating over multiple mini-batches. This setting approximates data-scarce or rapid-deployment scenarios with limited labelled examples. The layer-local learning rule of the Forward–Forward algorithm is well-suited to this regime. As each layer is optimised independently on a fixed batch, all layers can be updated in a single forward pass without revisiting the data. In contrast, standard backpropagation typically requires multiple mini-batch iterations for stable convergence. In the experiments, we demonstrate that performance can be reproduced at up to approximately 50% of the full-data level in the one-shot setting.

### 3.2 Supervised classification

**Figure 5:**
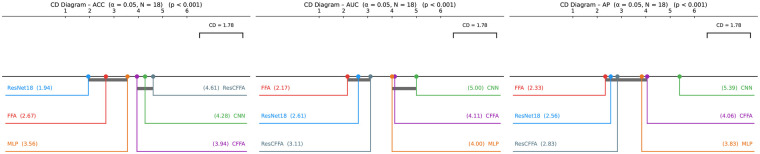
Critical Difference diagrams (Nemenyi post-hoc test, α=0.05, CD=1.78) across 18 MedMNIST datasets. Lower rank is better; methods connected by a grey bar are not significantly different. Significant differences are found for all metrics (Friedman p=0.0001/p<0.001/p<0.001 for ACC/AUC/AP). ResNet18 and FFA consistently rank among the top two, with CNN ranking last for AUC and AP.

Table 3 compares SaFF-AD against MLP, CNN, and ResNet18 across 2D and 3D benchmarks; Figure 5 summarises the statistical ranking across all 18 MedMNIST datasets. On MNIST, SaFF-AD with FFA achieves near-saturated performance, improving accuracy by approximately 5%–20% over MLP and CNN while using comparable or fewer parameters. Across MedMNIST 2D tasks, ResNet18 ranks best in accuracy on the majority of datasets, yet SaFF-AD with FFA or CFFA achieves the highest accuracy on OCT and Pneumonia, outperforming ResNet18 by up to 28% (OCT, CFFA vs. ResNet18: 0.703 vs. 0.421). In terms of AUC, the CD diagrams confirm FFA as the top-ranked method overall (avg. rank 2.17), significantly outperforming CNN (p<0.001). For AP, FFA, ResNet18, and ResCFFA form one indistinct top group, with CNN the sole significantly inferior method. On MedMNIST 3D, ResNet18 again leads in accuracy, while CFFA achieves the best result on Adrenal (0.772) and competitive performance on Organ and Synapse. A class-wise evaluation on every subset of MedMNIST can be found in Supplementary Tables S2 for 2D and S3 for 3D. For binary CXR classification, SaFF-AD matches near-perfect accuracy and AUC across all baselines. Overall, SaFF-AD offers a favourable trade-off: FFA and CFFA achieve top or near-top AUC and AP rankings with 14×–58× fewer parameters than ResNet18, making them compelling for resource-constrained medical imaging applications.

**Table 3:** Supervised Classification (Batchsize 60,000/MAX; Early Stopping, max 100 Epochs, Seed).

Dataset											SaFF-AD *(Ours)*								
		MLP			CNN			ResNet18			FFA			CFFA			ResCFFA		
		ACC↑	AUC↑	AP↑	ACC↑	AUC↑	AP↑	ACC↑	AUC↑	AP↑	ACC↑	AUC↑	AP↑	ACC↑	AUC↑	AP↑	ACC↑	AUC↑	AP↑
	MNIST	0.798	0.959	0.796	0.512	0.859	0.730	0.921	0.990	0.961	**0.970**	**0.999**	0.993	0.873	0.987	0.916	0.928	0.986	**0.993**
MedMNIST	Path	0.416	0.768	0.361	0.445	0.832	0.441	**0.632**	**0.912**	**0.580**	0.494	0.822	0.421	0.266	0.593	0.178	0.416	0.820	0.388
	Chest	0.638	0.524	0.080	**0.640**	0.492	0.073	0.635	0.541	0.082	0.412	**0.583**	0.085	0.052	0.542	0.085	0.253	0.559	**0.086**
	Derma	**0.669**	0.692	0.206	**0.669**	0.673	0.199	0.661	**0.805**	**0.322**	0.355	0.716	0.245	0.237	0.600	0.202	0.103	0.606	0.214
	OCT	0.356	0.624	0.373	0.250	0.522	0.280	0.421	0.708	0.466	0.626	0.854	0.658	**0.703**	**0.891**	0.679	0.634	0.873	**0.684**
	Pneum.	0.849	0.913	0.903	0.625	0.149	0.338	0.694	0.784	0.783	**0.888**	0.893	0.899	0.886	0.904	0.857	0.854	**0.923**	**0.920**
	Retina	0.477	0.642	0.335	0.435	0.657	0.312	**0.512**	0.679	0.357	0.463	0.694	0.354	0.388	0.656	0.320	0.405	**0.726**	**0.386**
	Breast	0.731	0.728	0.678	0.731	0.579	0.563	**0.859**	**0.855**	**0.794**	0.788	0.822	0.772	0.750	0.740	0.673	0.673	0.748	0.713
	Blood	0.447	0.829	0.445	0.170	0.628	0.285	**0.803**	**0.970**	**0.833**	0.756	0.949	0.759	0.541	0.828	0.510	0.495	0.906	0.598
	Tissue	0.392	0.567	0.158	0.317	0.615	0.170	**0.483**	0.747	0.308	0.472	**0.819**	**0.362**	0.341	0.708	0.260	0.341	0.612	0.192
	OrganA	0.434	0.860	0.461	0.292	0.756	0.329	**0.756**	**0.968**	**0.830**	0.588	0.878	0.496	0.282	0.737	0.249	0.369	0.835	0.441
	OrganC	0.402	0.853	0.565	0.223	0.676	0.192	**0.752**	**0.964**	**0.820**	0.636	0.895	0.552	0.256	0.637	0.195	0.393	0.847	0.432
	OrganS	0.306	0.782	0.361	0.235	0.635	0.165	**0.585**	**0.919**	**0.595**	0.453	0.855	0.390	0.279	0.643	0.199	0.345	0.821	0.394
MedMNIST3D	Organ	0.221	0.808	0.536	0.115	0.642	0.155	**0.634**	**0.926**	**0.707**	0.364	0.802	0.345	0.433	0.810	0.365	0.275	0.770	0.259
	Nodule	0.793	0.621	0.590	0.793	0.400	0.463	**0.814**	0.658	0.641	0.800	**0.770**	**0.671**	0.697	0.714	0.615	0.646	0.613	0.584
	Adrenal	0.768	**0.666**	0.609	0.768	0.474	0.481	0.768	0.515	0.510	0.607	0.660	**0.614**	**0.772**	0.572	0.560	0.721	0.646	0.605
	Fracture	0.388	**0.621**	**0.442**	0.375	0.593	0.415	**0.437**	0.534	0.357	0.388	0.557	0.391	0.375	0.500	0.333	0.328	0.608	0.416
	Vessel	**0.887**	0.447	0.491	**0.887**	0.476	0.488	**0.887**	0.522	0.509	0.733	**0.738**	**0.653**	0.623	0.520	0.511	0.531	0.529	0.516
	Synapse	**0.730**	0.421	0.474	**0.730**	0.490	0.499	0.726	0.487	0.492	**0.730**	**0.568**	**0.545**	**0.730**	0.521	0.517	0.723	0.527	0.516
Bin. CXR		0.999	0.999	0.500	0.986	0.978	0.500	0.898	0.895	0.500	0.899	0.961	0.500	0.999	0.999	0.500	0.999	0.999	0.500
	#Par.	2,797,010			1,554,954			11,183,694			max. 2,797,010			194,432			148,933		

*SaFF-AD* with FFA is used for 1D (flatten) input data and CFFA is used for 2D/3D input data. Bin. CXR—Binary Classification (Cardiomegaly) on VinDr-CXR.

ACC, Accuracy; AUC, Area Under the Receiver Operating characteristic; AP, Mean Average Precision.

**1st-ranked**, 2nd-ranked.

### 3.3 Supervised, semi-supervised, and self-supervised learning

Table 4 summarises classification performance across supervised (SL), semi-supervised (SemSL), and self-supervised (SSL) settings. In the fully supervised regime, SaFF-AD achieves competitive or best-in-class results across most benchmarks, matching or exceeding established methods with accuracy and AUC improvements of approximately 3%–10% on MNIST and MedMNIST tasks, while using substantially fewer parameters in the CFFA variant (up to 90% fewer). In the semi-supervised one-shot setting, SaFF-AD shows pronounced gains over prior SemSL methods, improving accuracy and AUC by roughly 15%–35% on challenging datasets such as Pneumonia, OCT, and OrganA, and achieving state-of-the-art performance on several benchmarks. Compared to FFA, CFFA consistently delivers higher accuracy and AUC in SemSL, with improvements of around 5%–20% while remaining significantly more parameter-efficient. Under self-supervised learning, SaFF-AD again performs strongly, with CFFA yielding accuracy gains of approximately 20%–40% over FFA on MedMNIST and near-saturated performance on binary VinDr-CXR. Overall, SaFF-AD demonstrates robust performance across learning paradigms, combining strong generalisation with substantial parameter efficiency gains of up to 90% compared to conventional architectures.

**Table 4:** Classification performance for Semi-supervised (SemSL), self-supervised (SSL), and average from established and our methods for fully supervised (SL).

Supervision	Method	#Par.			MedMNIST								VinDr-CXR	
			MNIST		Pneumonia		OCT		OrganA		Synapse3D		Binary	
			ACC↑	AUC↑	ACC↑	AUC↑	ACC↑	AUC↑	ACC↑	AUC↑	ACC↑	AUC↑	ACC↑	AUC↑
SL	∅	–	89.8	95.9	84.3	91.3	68.9	77.6	*78.7*	91.3	61.1	58.5	–	–
	Feurer et al. (54)	–	98.5	–	85.5	*94.2*	60.1	88.7	76.2	*97.6*	*73.0*	63.1	–	–
	FFA*	2.8m	*97.0*	*99.9*	*88.6*	91.2	62.6	86.0	58.9	87.8	27.0	23.8	89.9	96.1
	CFFA*	190k	87.3	98.7	87.0	90.0	*70.2*	*89.6*	33.6	77.1	*73.0*	*73.8*	*99.9*	*99.9*
SemSL	Zheng and Jia (55)	6m	–	–	56.6	79.9	**72.8**	69.9	–	–	–	–	–	–
	FFA (OS)*	2.8m	91.0	99.0	51.0	52.6	19.2	44.2	33.3	72.1	**73.0**	**74.8**	75.7	81.5
	CFFA (OS)*	190k	92.5	**99.2**	**86.2**	**93.1**	70.1	**89.5**	34.9	**80.2**	63.2	67.2	78.0	80.1
SSL	FFA*	2.8m	**92.9**	98.8	62.5	58.7	24.5	51.7	23.0	49.0	27.0	27.6	78.6	81.3
	CFFA*	190k	92.1	91.2	85.7	84.9	44.5	63.7	**58.2**	79.2	33.7	64.0	**99.2**	**99.6**

SaFF-AD with FFA is used for 1D (flatten) input data, and CFFA is used for 2D/3D input data.

OS, One-Shot; ACC, Accuracy; AUC, Area Under the Receiver Operating characteristic.

**1st-ranked**, 2nd-ranked, *Best for fully supervised*. ∅ (55–57); *Ours.

### 3.4 Pretrained models and downstream tasks

Table 5 evaluates pretrained SaFF-AD variants on downstream classification, reconstruction, and segmentation tasks. For classification, the FFA backbone achieves the strongest overall performance, outperforming CFFA and ResCFFA by approximately 5%–10% in accuracy on MNIST and by around 1%–2% on Pneumonia, indicating that higher-capacity models benefit more from transfer in discriminative tasks. In reconstruction, ResCFFA yields the best perceptual quality, improving SSIM and PSNR by roughly 3%–5% over FFA and CFFA on Blood data, while CFFA attains the highest PSNR on Pneumonia with gains of about 2%–3%. For segmentation on VinDr-CXR, FFA performs best, exceeding CFFA and ResCFFA by approximately 2%–15% in accuracy and up to 15% in IoU. Overall, pretrained SaFF-AD models transfer effectively across tasks, with FFA favouring classification and segmentation accuracy, while CFFA and ResCFFA provide competitive downstream performance at up to 95% fewer parameters.

**Table 5:** Pretrained *SaFF-AD* with FFA, CFFA and ResCFFA, evaluating on Downstream Task.

Method	#Par.	Classification${}^{a}$				Reconstruction				Segmentation	
		MNIST		Pneumonia		Blood		Pneumonia		VinDr-CXR	
		ACC↑	AUC↑	ACC↑	AUC↑	SSIM↑	PSNR↑	SSIM↑	PSNR↑	ACC↑	IoU↑
FFA	2.8m	**78.2**	**87.9**	**84.9**	**89.5**	$\underline{48.3}$	$\underline{16.7}$	72.3	$\underline{16.8}$	**93.2**	**40.1**
CFFA	190k	72.3	$\underline{86.5}$	84.0	88.7	$\underline{48.3}$	$\underline{16.7}$	$\underline{71.6}$	17.3	90.1	24.7
ResCFFA	150k	$\underline{73.4}$	85.2	$\underline{84.5}$	$\underline{89.2}$	**50.1**	**17.1**	63.8	16.3	$\underline{91.4}$	$\underline{25.8}$

ACC, Accuracy; AUC, Area Under the Receiver Operating characteristic; AP, Mean Average Precision; SSIM, Mean Structural Similarity Index; PSNR, Peak Signal-to-Noise Ratio; IoU, Intersection over union.

${}^{a}$with Triplet Margin Loss.

**1st-ranked**, 2nd-ranked.

### 3.5 Self-supervised anomaly detection

Supplementary Table S1 reports self-supervised anomaly detection results using Poisson Image Interpolation (17) across multiple input resolutions and anatomical regions. Experiments are conducted on the middle slice for 2D, serving as a conservative lower-bound comparison. SaFF-AD variants consistently achieve competitive AUC and AP scores relative to MLP and CNN baselines and, in several settings, match or exceed the performance of ResNet18-based models while requiring substantially fewer parameters (up to approximately 99% fewer in the most compact configurations). SaFF-AD supports training with either the task-specific anomaly loss $L_{\mathrm{anomaly}}$ or the more general representation loss $L_{\mathrm{general}}$. Across most configurations, $L_{\mathrm{anomaly}}$ yields stronger anomaly separation, reflected in higher AUC and AP. However, models trained with $L_{\mathrm{general}}$ occasionally achieve comparable or improved performance, particularly at intermediate or higher resolutions, suggesting that the learned representations can generalise beyond the specific anomaly objective. Residual CFFA variants exhibit robust performance at higher resolutions, consistent with the stabilising effect of residual and multi-scale feature propagation. For Abdomen data, 2D CFFA models achieve the strongest overall results among SaFF-AD variants, while the choice of loss function has a moderate but resolution-dependent impact. One-shot variants, while highly parameter-efficient, generally underperform fully trained counterparts, highlighting the importance of iterative layer-wise optimisation.

Across anomaly types, performance varies substantially. For the Abdomen subset (Table 6), split morphology (test2) is the most reliably detected anomaly, with CE-based methods achieving AUC above 0.95 with MLP (CE: 0.9962, CE+Mahal.: 0.9856) and ResNet18 (CE+Mahal.: 0.9741), and above 0.85 with CNN. Added texture (test1) is strongly detected when Mahal.-based methods are paired with MLP (EBM+Mahal.: 0.92, CE+Mahal.: 0.91), but performance is more moderate for CNN and ResNet18 backbones (AUC up to 0.79). Abnormal organ location (test3) is detected well by CNN-based models (AUC up to 0.87 with CE+Mahal.) but remains harder for MLP and ResNet18. Complete organ absence (test4) is generally the most challenging case; however, FF with CNN clearly outperforms all other configurations, achieving AUC 0.72, while remaining methods stay near chance level.

**Table 6:** Self-supervised Anomaly detection results on AADD for input size 64×64, for subset Abdomen. Normal test set (test0) against Abnormal test sets (test1–4).

Dataset			EBM		EBM + Mahal.		CE		CE + Mahal.		FF (*Ours*)	
	Model	Subset	AUC↑	AP↑	AUC↑	AP↑	AUC↑	AP↑	AUC↑	AP↑	AUC↑	AP↑
Abdomen	MLP	test1	0.5869	0.2760	**0.9209**	**0.8215**	0.7476	0.4442	0.9085	0.6750	0.4890	0.1948
		test2	0.5877	0.2778	0.7810	0.3801	**0.9962**	**0.9905**	0.9856	0.9541	0.5413	0.2355
		test3	0.4471	0.1792	0.5631	0.2356	0.4199	0.1647	0.5701	0.2516	**0.6129**	**0.2605**
		test4	0.5965	**0.3128**	0.5085	0.2067	0.5299	0.2267	0.5929	0.2655	**0.6041**	0.2376
	CNN	test1	0.4385	0.1705	0.6017	0.3082	0.4568	0.1952	**0.6885**	**0.3143**	0.6261	0.2713
		test2	0.5098	0.1941	0.8553	0.5937	0.6958	0.3343	**0.8603**	**0.6269**	0.7135	0.3149
		test3	0.4780	0.2067	0.8346	0.5496	0.8583	**0.6560**	**0.8683**	0.6430	0.7044	0.3137
		test4	0.5156	0.2236	0.5475	0.2434	0.5407	0.2252	0.5199	0.2241	**0.7246**	**0.3276**
	ResNet18	test1	0.5331	0.2225	**0.7889**	**0.6106**	0.5508	0.2689	0.6632	0.2886	0.4982	0.2421
		test2	0.7426	0.3787	0.8726	0.5945	0.9013	0.7883	**0.9741**	**0.9028**	0.4555	0.1871
		test3	0.6020	0.2298	**0.6238**	**0.2927**	0.5072	0.2008	0.5236	0.2032	0.4063	0.1719
		test4	**0.5657**	**0.2593**	0.5434	0.2096	0.5149	0.2165	0.5553	0.2328	0.3130	0.1801

AUC, Area Under the Receiver Operating characteristic; AP, Mean Average Precision.

**1st-ranked**, 2nd-ranked.

For the Brain subset (Table 7), over-rotation (test1) is the most consistently difficult anomaly, with most AUC scores below 0.60 and no method exceeding 0.67. Under-rotation (test2) is more detectable in select configurations: EBM with ResNet18 achieves 0.94, and FF reaches 0.78 with MLP, the best result among all methods for that backbone. Globally (test3) and regionally (test4) added spatial frequencies are moderately detectable, with CE+Mahal. with MLP yielding the highest AUC values (0.73 and 0.71, respectively), and CE with ResNet18 reaching 0.71 on test3. No single method or backbone dominates consistently; CE+Mahal. tends to be the most reliable overall, while FF shows notably strong performance on test2 with MLP.

**Table 7:** Self-supervised Anomaly detection results on AADD for input size 64×64, for subset Brain. Normal test set (test0) against Abnormal test sets (test1–4).

Dataset			EBM		EBM + Mahal.		CE		CE + Mahal.		FF (*Ours*)	
	Model	Subset	AUC↑	AP↑	AUC↑	AP↑	AUC↑	AP↑	AUC↑	AP↑	AUC↑	AP↑
Brain	MLP	test1	0.1779	0.1224	0.5403	**0.2600**	0.5359	0.2319	**0.5541**	0.2459	0.3788	0.1522
		test2	0.4693	0.1873	0.5740	0.2596	0.5151	0.2325	0.5943	0.2929	**0.7774**	**0.3869**
		test3	0.6304	0.3238	0.5847	0.2470	0.6613	0.3191	**0.7288**	**0.4187**	0.5336	0.2105
		test4	0.6145	0.3405	0.4425	0.1764	0.6661	0.3346	**0.7127**	**0.4345**	0.4711	0.2048
	CNN	test1	0.5483	0.2158	0.4835	0.2170	0.5435	0.3195	**0.6674**	**0.3355**	0.6180	0.2915
		test2	**0.7220**	**0.4476**	0.5754	0.2529	0.5133	0.1965	0.5620	0.2379	0.5243	0.2381
		test3	**0.7222**	**0.3889**	0.6828	0.3716	0.5765	0.3229	0.6533	0.3540	0.5703	0.2586
		test4	0.5235	0.2123	0.7192	0.4221	0.4508	0.2357	**0.7603**	**0.4435**	0.5198	0.2210
	ResNet18	test1	0.3588	0.1689	**0.5899**	**0.2914**	0.5295	0.2289	0.5876	0.2276	0.4351	0.1875
		test2	**0.9417**	**0.7753**	0.5826	0.2616	0.4911	0.1969	0.5077	0.2062	0.7588	0.5321
		test3	0.5719	0.2647	0.6477	0.2951	**0.7082**	**0.4009**	0.6435	0.2855	0.4959	0.2317
		test4	0.5297	0.2303	**0.7170**	**0.3982**	0.6767	0.3970	0.6556	0.2848	0.4743	0.2027

AUC, Area Under the Receiver Operating characteristic; AP, Mean Average Precision.

**1st-ranked**, 2nd-ranked.

**Figure 6:**
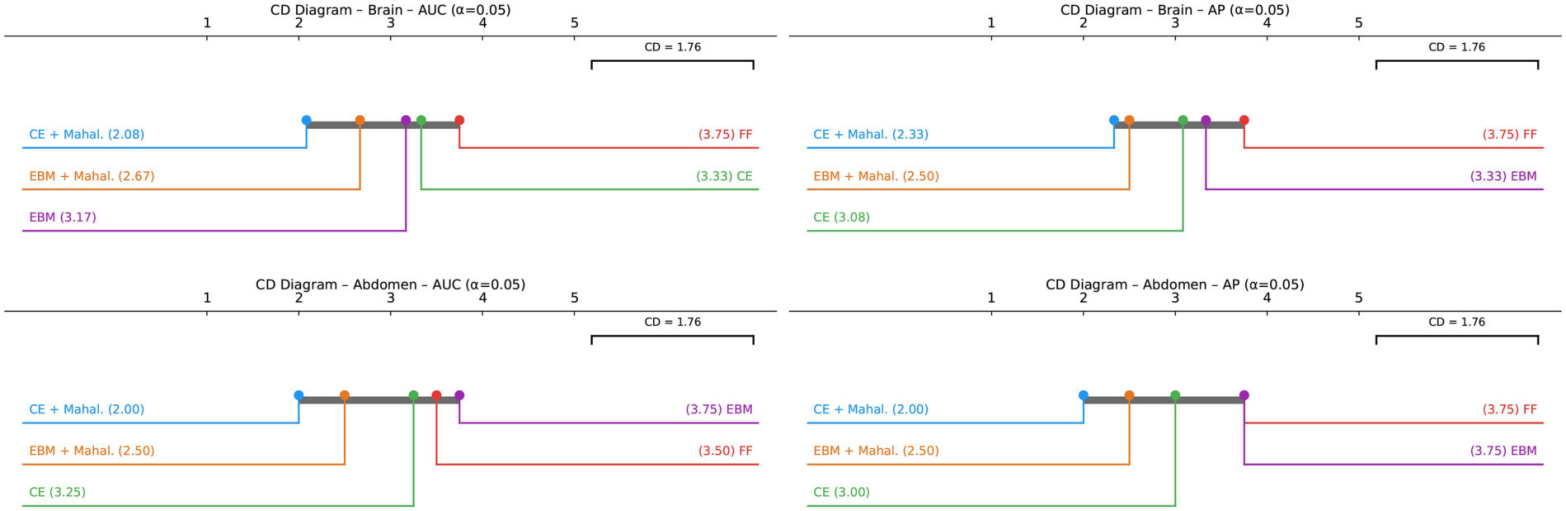
Critical Difference diagrams (Nemenyi post-hoc test, α=0.05) for the AADD dataset at input size 64×64, comparing anomaly detection methods across all models (MLP, CNN, ResNet18) and abnormal test sets (test1–4). Rankings are shown separately for AUC (left column) and Average Precision (right column), for the Brain subset (top row) and Abdomen subset (bottom row). Lower rank indicates better performance; methods connected by a grey bar are not significantly different from one another. For the Brain subset, no statistically significant differences are found (Friedman p=0.094 for AUC, p=0.159 for AP). For the Abdomen subset, the Friedman test reaches significance (p=0.037 for AUC, p=0.022 for AP), yet no individual pairwise comparisons survive the Nemenyi post-hoc correction (CD=1.76).

Overall, performance varies only moderately across resolutions, indicating that SaFF-AD is relatively robust to input size. Statistical analysis via Nemenyi post-hoc tests confirms that no method achieves a statistically significant pairwise advantage over others, see Figure 6. For the Brain subset, the Friedman test finds no significant overall difference (Friedman p=0.094 for AUC, p=0.159 for AP). For the Abdomen subset, the Friedman omnibus test reaches significance (p=0.037 for AUC, p=0.022 for AP); however, no individual pairwise comparisons survive the Nemenyi post-hoc correction (CD=1.76), indicating that while a global ranking signal exists, no single method consistently dominates another. These results demonstrate that SaFF-AD provides a flexible and parameter-efficient framework for self-supervised anomaly detection, balancing task-specific optimisation with general representation learning across different data domains.

### 3.6 Scaling and efficiency

**Figure 7:**
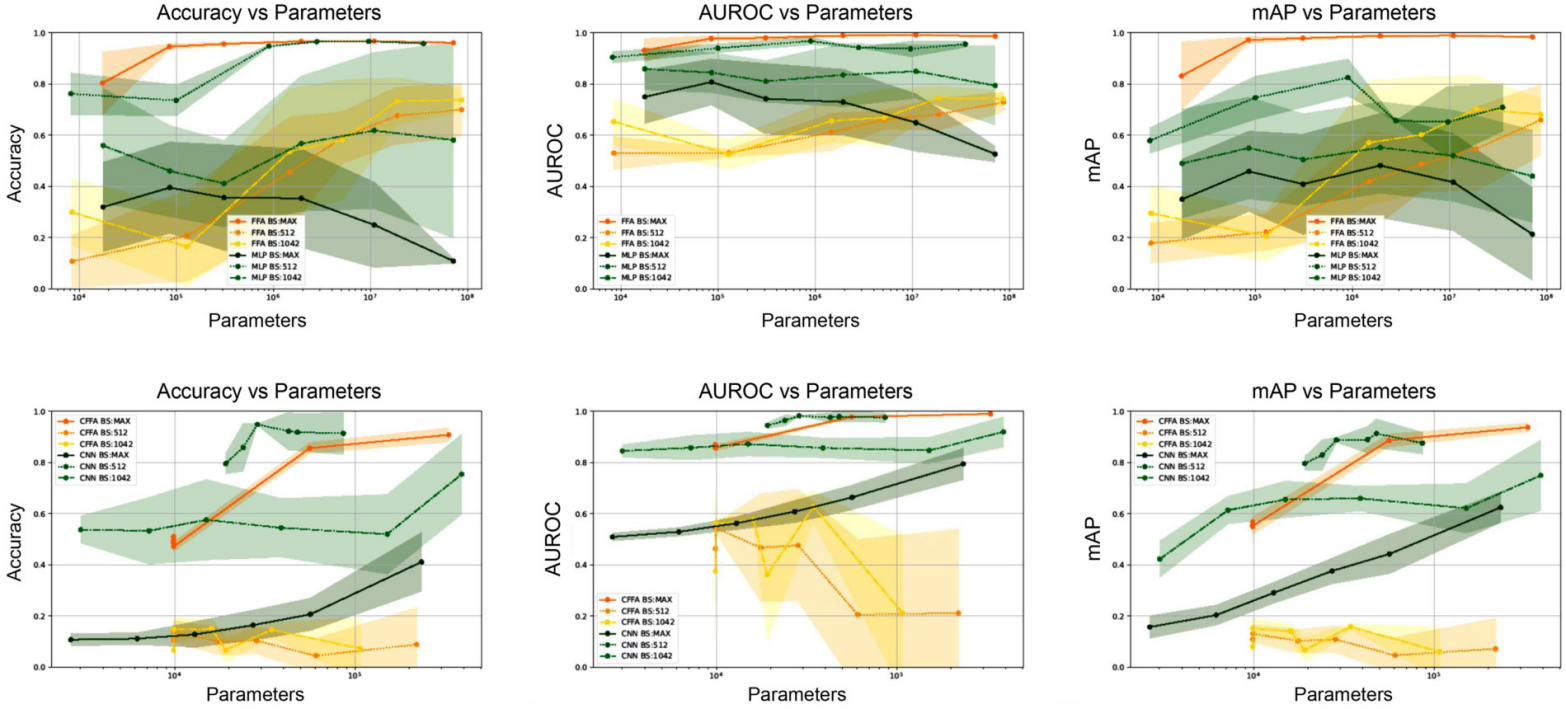
Classification on MNIST. ACC, Accuracy; AUC, Area Under the Receiver Operating characteristic; mAP, Mean Average Precision vs. Number of Parameters Comparison with maximum batch size. MLP vs. FFA (top), CNN vs. CFFA (bottom). *Ours* in orange.

The scaling and efficiency evaluation in Figure 7 demonstrates that SaFF-AD exhibits favourable computational scaling as model size and data complexity increase. Across settings, SaFF-AD reduces the number of function evaluations during training and overall training cost by approximately 40%–80% compared to conventional baselines, while maintaining comparable or improved predictive performance. Parameter-efficient variants such as CFFA and ResCFFA scale particularly well, achieving up to 90% fewer parameters with only marginal performance degradation, and in several cases even improving accuracy and AUC by around 2%–5%. Overall, these results highlight that SaFF-AD offers a strong accuracy-efficiency trade-off, enabling scalable training and deployment across 1D, 2D, and 3D tasks under constrained computational budgets.

## 4 Discussion

SaFF-AD can handle 1D, 2D, and 3D data across supervised, semi-supervised, and self-supervised learning paradigms. Extensive ablation studies reveal distinct characteristics of the different backbones. The FFA backbone achieves substantial gains, particularly in one-shot scenarios, with accuracy improvements of several hundred percent and computational reductions of up to 90%. Early stopping and training thresholds provide the most favourable trade-offs, enabling significant efficiency gains without compromising performance, whereas aggressive pruning or warm-up can lead to severe degradation. In contrast, the CFFA backbone exhibits more modest accuracy gains (a few percent) but delivers stable performance across resolutions, with computational savings of up to ∼50%, reflecting a reliable accuracy-efficiency balance. Residual CFFA (ResCFFA) further stabilises high-resolution performance, particularly in complex 3D medical imaging tasks. In fully supervised classification, SaFF-AD consistently outperforms MLP and CNN baselines, achieving accuracy and AUC improvements of 5%–30% on challenging datasets while remaining competitive with ResNet18 at substantially lower parameter cost. In semi-supervised one-shot settings, CFFA variants show pronounced gains of 15%–35%, illustrating strong generalisation under limited labelled data. Self-supervised learning similarly benefits from the CFFA backbone, with gains of 20%–40% on MedMNIST and near-saturated performance on binary VinDr-CXR, demonstrating that SaFF-AD can effectively learn robust representations even without explicit labels. Self-supervised anomaly detection constitutes a central capability of SaFF-Net. Across anatomical regions and input resolutions, SaFF-AD variants consistently achieve competitive performance relative to MLP and CNN baselines and, in several configurations, match or exceed ResNet18-based models while requiring substantially fewer parameters (up to approximately 99% fewer in compact settings). Training with the task-specific loss $L_{\text{anomaly}}$ generally improves anomaly separability, whereas $L_{\text{general}}$ promotes representations that can remain robust at intermediate and higher resolutions. Residual CFFA variants demonstrate stable performance as resolution increases, and 2D CFFA models achieve strong results on both Brain and Abdomen datasets. These findings highlight SaFF-AD’s ability to efficiently detect subtle anomalies while balancing parameter efficiency and representational robustness.

The nature of the anomaly cases further informs the interpretation of these results. For the Abdomen subset, split morphology (test2) is the most reliably detected anomaly across all backbones, with CE-based methods achieving near-perfect AUC in several configurations, likely because the structural discontinuity introduced by splitting introduces highly discriminative local patterns that are captured well by both spatial and feature-space scoring. Added organ texture (test1) is strongly detected when Mahalanobis-based scoring is combined with MLP, suggesting that texture deviations manifest as distributional outliers in the learned feature space; however, the advantage diminishes for CNN and ResNet18 backbones, possibly due to their stronger spatial inductive biases suppressing texture-specific signals. Abnormal organ location (test3) is handled best by CNN-based models, consistent with their sensitivity to spatial structure, while MLP and ResNet18 struggle more. Notably, complete organ absence (test4) is generally the most challenging case, yet FF with CNN achieves AUC 0.72, clearly outperforming all other method-backbone combinations on this anomaly type. This may reflect FF’s reliance on positive-negative signal contrast rather than feature reconstruction, making it less dependent on the presence of distinctive structure.

For the Brain subset, the anomaly cases are more naturally framed as out-of-distribution detection problems centred on frequency-domain or orientation-based deviations. Over-rotation (test1) and under-rotation (test2) alter the global orientation statistics of the input, while globally added spatial frequencies (test3) and regionally added spatial frequencies (test4) introduce higher-frequency content either across the entire image or within a localised region. Over-rotation (test1) is the most consistently difficult anomaly, with no configuration exceeding AUC 0.67, likely because the deviation in orientation statistics is subtle relative to the within-class variability of normal samples. Under-rotation (test2) shows considerably higher but uneven detectability: EBM with ResNet18 achieves AUC 0.94, and FF with MLP reaches 0.78, the strongest result among all MLP configurations, while other combinations remain near chance. This variability suggests that sensitivity to global orientation changes is strongly dependent on the interaction between the scoring objective and the backbone’s representational inductive biases. Test3 and test4 are moderately detectable, with CE+Mahal. With MLP yielding the strongest results (0.73 and 0.71, respectively), suggesting that Mahalanobis-based scoring may be better suited to capturing distributional shifts in frequency space. FF notably outperforms other methods on test2 with MLP, possibly reflecting sensitivity to global orientation changes in lower-dimensional feature spaces.

Scaling and efficiency analyses further demonstrate that SaFF-AD maintains high predictive performance while substantially reducing computational cost, achieving 40%–80% fewer function evaluations across tasks. Parameter-efficient variants such as CFFA and ResCFFA scale effectively, with only marginal performance degradation despite up to 90% fewer parameters. These properties make SaFF-AD particularly well-suited for deployment on resource-constrained platforms, including embedded and neuromorphic systems, without sacrificing accuracy or robustness. From a clinical perspective, SaFF-AD’s combination of high accuracy in classification and anomaly detection, and computational efficiency suggests strong potential for real-time diagnostic and monitoring applications. Its ability to generalise across 1D, 2D, and 3D modalities makes it adaptable to diverse imaging protocols, while its low parameter footprint and efficient scaling enable integration into low-power devices, edge computing, and neuromorphic hardware.

### 4.1 Limitations and future work

Anomaly detection experiments on AADD are conducted on individual 2D slices, offering a conservative lower-bound evaluation that nonetheless demonstrates robustness without full volumetric context; extending to 3D inputs is a natural next step that may yield further performance gains. Self-supervised anomaly generation via PII and patch blending produces realistic pseudo-anomalies and establishes a strong training signal, yet sensitivity to rarer or structurally atypical pathologies that deviate from the synthetic prior remains an open challenge. Richer augmentation strategies and task-adaptive anomaly synthesis are therefore promising directions for improving coverage of the anomaly space.

Training uses Poisson image interpolation (PII) only to introduce local inconsistencies and mild rotation by 10 degrees, which means expanding [30,60] degree rotation for normal to [20,70] degrees, while the AADD defines anomalies through rotations (over-rotation [75–105] degrees and under-rotation [0–15]), spatial frequency changes, and structural modifications like missing organs. This ensures that the model is not explicitly fitted to the test-time anomalies. Instead, it learns to detect general deviations in spatial and anatomical coherence. PII introduces diverse, non-deterministic irregularities that do not directly match the evaluation anomalies, preserving meaningful separation between training and testing and supporting a realistic open-world setting.

SaFF-AD’s self-configuration substantially reduces manual hyperparameter tuning, though the warm-up phase introduces additional function evaluations and can affect stability in certain configurations; refining the warm-up selection criteria to be more principled and data-adaptive would further strengthen robustness across diverse modalities. Evaluations have so far been conducted on NVIDIA A100 GPUs, hence, empirical validation of energy consumption and inference latency on such hardware remains an important direction for future work. CFFA variants achieve competitive classification performance across MedMNIST, with a moderate accuracy gap relative to ResNet-18 on some multi-class tasks, motivating further exploration of capacity-efficient architectural designs within the forward-forward paradigm. All experiments employ standardised preprocessing pipelines, providing a controlled baseline; future work should assess robustness under heterogeneous acquisition conditions, varying scanner vendors, and diverse patient populations to establish clinical deployability.

Looking ahead, applying SaFF-AD to dynamic and temporal imaging modalities, such as video sequences and longitudinal studies, would probe its ability to capture temporal anomalies and distributional drift over time. Efficient adaptation for embedded and neuromorphic systems remains a central goal, with the prospect of enabling real-time, low-power deployment in clinical environments. Developing more expressive and theoretically grounded loss functions could further improve anomaly separability and representational robustness across resolutions and domains. Finally, integration into clinical workflows, for rapid anomaly flagging, diagnostic support, and patient monitoring, represents the ultimate translational objective and will require prospective evaluation in real-world settings.

## 5 Conclusion

This work revisited back-propagation-free learning as a principled and resource-efficient paradigm for anomaly and out-of-distribution detection in medical imaging. We discussed the Convolutional Forward-Forward Algorithm, demonstrating that convolutional structure and layer-wise local objectives overcome key scalability limitations of existing forward-forward approaches while preserving their computational advantages. A central finding is that the forward-forward goodness signal constitutes an intrinsic and interpretable measure of conformity to the learned data distribution, enabling self-supervised anomaly detection without auxiliary networks, post-hoc uncertainty estimation, or heuristically designed scoring functions. Building on this insight, we introduced SaFF-AD, a self-adaptive forward-forward network that jointly optimises representation learning, architectural configuration, and anomaly discrimination under resource constraints. Through adaptive depth selection, trainable threshold coupling, structured pruning, and layer-wise normalisation, SaFF-AD achieves stable learning across one-shot and full training regimes, supervised and self-supervised settings, and 1D, 2D, and 3D imaging modalities. Extensive experiments across MNIST, MedMNIST, VinDr-CXR, and the AADD benchmark demonstrate that SaFF-AD delivers competitive or superior performance relative to backpropagation-trained models, including ResNet-18, while requiring substantially fewer parameters and forward evaluations. Anomaly detection results on the AADD benchmark further reveal an important distinction between medical anomaly detection and OOD detection. Structurally salient anomalies, such as morphological deformations or abnormal organ textures, are detected more reliably across all methods, as they introduce distinctive patterns that diverge clearly from the learned normal distribution. In contrast, frequency-domain deviations, such as subtle global or regional spectral perturbations, present a fundamentally harder OOD detection problem, with performance remaining near chance level across all evaluated approaches. This distinction underscores the importance of carefully characterising the nature of expected anomalies when designing and evaluating detection systems, and highlights frequency-based OOD detection as an open challenge for future work. These results establish forward-forward learning as a viable and practically attractive alternative to conventional deep learning for safety-critical medical image analysis, particularly in settings characterised by constrained computational budgets, limited labelled data, and distributional uncertainty. By treating anomaly detection as an intrinsic property of the learned model rather than a post-hoc addition, SaFF-AD offers a unified framework that is interpretable, efficient, and well-suited to the open-world conditions encountered in real-world clinical deployment.

## Data Availability

The original contributions presented in the study are included in the article/**Supplementary Material**, further inquiries can be directed to the corresponding author.

## Footnotes

^1^Code: https://github.com/ividja/SaFF-AD
