# Supplementary material for: Self-adaptive forward-forward network for anomaly detection and medical image analysis
Source: Front Radiol. 2026 Jun 11;6:1771850. doi: 10.3389/fradi.2026.1771850 (PMC13294442; doi:10.3389/fradi.2026.1771850)
Supplement: Supplementary file 1 [file Datasheet1.pdf]

Supplementary Material

BASELINE ARCHITECTURES

Multi-Layer Perceptron (MLP)

The MLP baseline flattens the input image into a one-dimensional vector and processes it through a sequence of fully connected layers, each followed by a ReLU activation. In all experiments, three hidden layers of width 1000 are used, forming a four-layer architecture that maps from the input dimension d_{in} to C output classes. No batch normalisation or dropout is applied. The model is trained end-to-end using cross-entropy loss with the Adam optimiser and a learning rate of $3{\times}10^{-4}$.

Convolutional Neural Network (CNN)

The two-dimensional CNN consists of four convolutional blocks followed by global average pooling and a linear classification head. Each block comprises a convolutional layer, a max-pooling operation (kernel size 2, stride 2, padding 1), and a ReLU activation. The number of filters increases across blocks as 64, 128, 256, and 512, with all convolutions using $3{\times}3$ kernels and no padding. The first block applies a stride of 2, while subsequent blocks use a stride of 1. After the convolutional stages, global average pooling reduces the spatial dimensions to $1{\times}1$, and a final linear layer produces class logits for C categories.

The three-dimensional variant replaces each two-dimensional operation with its volumetric counterpart (`Conv3d`, `MaxPool3d`, `AdaptiveAvgPool3d`) while retaining the same filter progression of 64, 128, 256, and 512, stride schedule, and kernel size. Neither the two-dimensional nor the three-dimensional model incorporates batch normalisation or dropout. Both are trained using cross-entropy loss and the Adam optimiser with a learning rate of $3{\times}10^{-5}$.

ResNet-18

The ResNet-18 baseline follows the standard torchvision configuration, comprising four residual stages with depths $[2, 2, 2, 2]$ and channel widths $[64, 128, 256, 512]$. The network is initialised from scratch without ImageNet pre-training. The final fully connected layer is replaced with a linear projection from 512 features to C output classes. As several datasets considered in this study are greyscale, single-channel inputs are replicated across three channels prior to being passed through the network. The model is trained using cross-entropy loss and the Adam optimiser with a learning rate of $3{\times}10^{-5}$.

ACTIVATION REGULARISATION

For fully connected and three-dimensional convolutional layers, a *peer normalisation* penalty is introduced to prevent inactive or saturated neurons. A running mean of layer activations is maintained via an exponential moving average with momentum 0.9:

$$\bar{\mu}t = 0.9, \bar{\mu}t - 1 + 0.1, \mu_t, \tag{S1}$$

and the regularisation term $\mathcal{L}_{\text{peer}} = \overline{(\mu_t - \bar{\mu}_t)^2}$ is added to the layer loss with weight $\lambda = 0.03$.

Table S1. Self-Supervised Anomaly Detection with Poisson Image Interpolation Tan et al. (2021) (Batch size 60,000/MAX; early stopping, max 100 epochs, seed). *SaFF-AD* with FFA is used for 1D (flattened) input data and CFFA for 2D and 3D input data. * indicates training with $\mathcal{L}_{\text{general}}$ instead of $\mathcal{L}_{\text{anomaly}}$. OS - One-Shot. AUC - Area Under the ROC Curve, AP - Average Precision. **1st-ranked**, 2nd-ranked.

								SaFF-AD *(Ours)*									
								2D								3D	
		MLP		CNN		ResNet18		FFA		CFFA		CFFA*		ResCFFA		CFFA (OS)		CFFA* (OS)	
	**AADD**	AUC↑	AP↑	AUC↑	AP↑	AUC↑	AP↑	AUC↑	AP↑	AUC↑	AP↑	AUC↑	AP↑	AUC↑	AP↑	AUC↑	AP↑	AUC↑	AP↑
Brain	28×28	0.58	**0.65**	0.50	0.50	0.53	0.56	0.58	0.63	0.51	0.54	0.50	0.50	0.51	0.50	**0.92**	0.50	0.22	0.50
	64×64	0.54	0.58	0.45	0.43	**0.58**	0.56	0.50	0.46	0.50	0.56	0.50	0.50	**0.58**	**0.63**	0.48	0.50	0.21	0.50
	96×96	0.55	**0.59**	0.50	0.50	**0.56**	**0.59**	0.51	0.52	0.50	0.54	0.47	0.51	0.52	0.52	0.23	0.50	0.52	0.50
	128×128	0.52	**0.56**	0.41	0.39	**0.58**	0.55	0.53	0.54	0.50	**0.56**	0.50	0.50	0.51	0.52	0.24	0.50	0.14	0.50
Abdomen	28×28	0.58	0.65	0.40	0.40	0.50	0.52	0.61	0.68	0.36	0.38	0.61	0.61	0.40	0.40	0.46	0.50	**0.73**	0.50
	64×64	0.54	0.58	0.47	0.48	0.61	**0.61**	0.50	0.46	0.51	0.50	0.61	0.60	0.43	0.41	**0.66**	0.50	0.20	0.50
	96×96	0.55	0.59	0.61	0.68	0.56	0.63	0.50	0.53	**0.73**	**0.73**	0.52	0.52	0.52	0.55	0.55	0.50	0.20	0.50
	128×128	0.52	**0.56**	0.48	0.50	**0.55**	**0.56**	0.50	0.47	0.50	0.50	0.52	0.50	0.44	0.40	0.36	0.50	0.14	0.50
	#Par.	2,797,010		1,554,954		11,183,694		max. 2,797,010		370,243		370,243		148,933		2,326,532		2,326,532	

For two-dimensional convolutional layers, this is replaced by a batch normalisation loss that penalises deviation of the activation distribution from zero mean and unit variance, with a smaller weight of $\lambda = 3{\times}10^{-5}$.

EARLY STOPPING

Training employs a three-level early stopping scheme operating at the granularity of (i) epochs, (ii) per-layer iterations, and (iii) layers. At each level, training is halted if the monitored validation loss fails to decrease by more than a minimum delta δ for a configurable number of patience steps.

Default values are: patience $= 5$ epochs at the epoch level, 10 iterations at the layer-iteration level, and 2 at the layer level, all with $\delta = 10^{-18}$. Fine-tuning, when performed, uses a learning rate reduced by a factor of 10^{-3} relative to the initial value.

CALIBRATION AND POST-PROCESSING

No explicit calibration procedure, such as temperature scaling or Platt scaling, is applied. Raw goodness scores are converted to class probabilities solely via standard softmax normalisation, which is used during evaluation to compute probability-based metrics such as AUROC. Predicted class labels are obtained directly as the argmax of the per-class goodness vector; no post-processing, such as label smoothing, ensembling, or threshold adjustment, is performed after training.

Calibration may nevertheless be important in clinical deployment, where overconfident probability estimates can mislead downstream decision-making. As the goodness scores are not trained to correspond directly to posterior probabilities, the raw softmax outputs are likely to be poorly calibrated, particularly on class-imbalanced datasets such as VinDr-CXR or ChestMNIST.

Temperature scaling, dividing logits by a scalar T fitted on a held-out validation set, is a natural and lightweight remedy: replacing $\mathrm{softmax}(g/T)$ for $T > 1$ reduces overconfident predictions towards the uniform distribution without altering the predicted class, making it readily compatible with the layer-wise Forward-Forward training scheme. Investigating the calibration of goodness-based classifiers and the suitability of standard recalibration methods remains an open direction for future work.

Table S2. Per-class AUROC / AP across datasets and model types (2D datasets). AUC - Area Under the Receiver Operating characteristic, AP - Mean Average Precision. Best checkpoint selected by highest Overall Accuracy.

					SaFF-AD *(Ours)*		
Dataset	Class	**MLP** (AUROC/AP)	**CNN** (AUROC/AP)	**ResNet18** (AUROC/AP)	**FFA** (AUROC/AP)	**CFFA** (AUROC/AP)	**ResCFFA** (AUROC/AP)
**Path**	adipose	1.00 / 0.98	0.99 / 0.96	0.98 / 0.90	0.98 / 0.91	0.42 / 0.19	0.94 / 0.77
	background	1.00 / 1.00	1.00 / 1.00	0.99 / 0.86	1.00 / 0.99	0.48 / 0.12	0.93 / 0.49
	debris	0.65 / 0.06	0.82 / 0.23	0.79 / 0.19	0.82 / 0.20	0.67 / 0.12	0.78 / 0.20
	lymphocytes	0.57 / 0.11	0.68 / 0.37	0.93 / 0.46	0.69 / 0.15	0.51 / 0.09	0.87 / 0.49
	mucus	0.81 / 0.30	0.70 / 0.20	0.94 / 0.84	0.67 / 0.37	0.77 / 0.37	0.82 / 0.43
	smooth muscle	0.72 / 0.14	0.79 / 0.22	0.94 / 0.54	0.77 / 0.21	0.53 / 0.09	0.68 / 0.11
	normal colon mucosa	0.65 / 0.13	0.87 / 0.31	0.86 / 0.38	0.89 / 0.40	0.60 / 0.12	0.77 / 0.20
	cancer-associated stroma	0.69 / 0.09	0.75 / 0.15	0.91 / 0.45	0.73 / 0.15	0.50 / 0.05	0.66 / 0.08
	colorectal adenocarcinoma epithelium	0.83 / 0.45	0.88 / 0.55	0.88 / 0.59	0.84 / 0.41	0.85 / 0.45	0.93 / 0.73
**Chest**	atelectasis	0.52 / 0.65	0.52 / 0.64	0.56 / 0.68	0.60 / 0.70	0.61 / 0.72	0.59 / 0.71
	cardiomegaly	0.48 / 0.02	0.50 / 0.02	0.59 / 0.03	0.67 / 0.04	0.68 / 0.04	0.67 / 0.04
	effusion	0.64 / 0.14	0.50 / 0.08	0.63 / 0.13	0.63 / 0.13	0.66 / 0.14	0.68 / 0.15
	infiltration	0.57 / 0.15	0.56 / 0.14	0.57 / 0.15	0.57 / 0.15	0.56 / 0.14	0.58 / 0.15
	mass	0.53 / 0.03	0.47 / 0.03	0.49 / 0.03	0.52 / 0.03	0.53 / 0.03	0.54 / 0.03
	nodule	0.58 / 0.04	0.46 / 0.03	0.48 / 0.03	0.55 / 0.04	0.55 / 0.04	0.57 / 0.04
	pneumonia	0.50 / 0.00	0.53 / 0.00	0.57 / 0.01	0.54 / 0.00	0.45 / 0.00	0.48 / 0.00
	pneumothorax	0.57 / 0.03	0.49 / 0.02	0.54 / 0.03	0.48 / 0.02	0.58 / 0.03	0.55 / 0.03
	consolidation	0.63 / 0.02	0.63 / 0.02	0.64 / 0.02	0.67 / 0.02	0.40 / 0.01	0.53 / 0.01
	edema	0.62 / 0.01	0.72 / 0.01	0.75 / 0.01	0.73 / 0.01	0.39 / 0.01	0.57 / 0.01
	emphysema	0.55 / 0.01	0.50 / 0.01	0.49 / 0.01	0.55 / 0.01	0.51 / 0.01	0.52 / 0.01
	fibrosis	0.41 / 0.01	0.32 / 0.01	0.36 / 0.01	0.56 / 0.01	0.55 / 0.01	0.50 / 0.01
	pleural	0.42 / 0.01	0.40 / 0.01	0.43 / 0.01	0.53 / 0.01	0.49 / 0.01	0.51 / 0.01
	hernia	0.31 / 0.00	0.28 / 0.00	0.47 / 0.00	0.56 / 0.00	0.61 / 0.00	0.54 / 0.00
**Derma**	actinic keratoses and intraepithelial carcinoma	0.65 / 0.04	0.71 / 0.06	0.81 / 0.23	0.82 / 0.11	0.81 / 0.12	0.86 / 0.13
	basal cell carcinoma	0.79 / 0.12	0.78 / 0.14	0.86 / 0.26	0.85 / 0.25	0.58 / 0.06	0.77 / 0.12
	benign keratosis-like lesions	0.73 / 0.23	0.74 / 0.30	0.82 / 0.40	0.60 / 0.19	0.69 / 0.21	0.74 / 0.24
	dermatofibroma	0.64 / 0.02	0.75 / 0.02	0.79 / 0.10	0.75 / 0.04	0.40 / 0.01	0.34 / 0.01
	melanoma	0.62 / 0.13	0.40 / 0.09	0.80 / 0.31	0.74 / 0.22	0.61 / 0.16	0.39 / 0.08
	melanocytic nevi	0.74 / 0.88	0.67 / 0.74	0.84 / 0.92	0.77 / 0.85	0.78 / 0.85	0.82 / 0.90
	vascular lesions	0.66 / 0.02	0.65 / 0.03	0.72 / 0.03	0.49 / 0.06	0.33 / 0.01	0.33 / 0.01
**OCT**	choroidal neovascularization	0.65 / 0.36	0.65 / 0.38	0.80 / 0.58	0.85 / 0.67	0.92 / 0.75	0.95 / 0.86
	diabetic macular edema	0.72 / 0.46	0.50 / 0.27	0.66 / 0.36	0.90 / 0.76	0.94 / 0.80	0.87 / 0.68
	drusen	0.40 / 0.20	0.52 / 0.27	0.54 / 0.27	0.77 / 0.45	0.82 / 0.53	0.76 / 0.44
	normal	0.73 / 0.47	0.42 / 0.20	0.83 / 0.65	0.90 / 0.76	0.89 / 0.63	0.91 / 0.76
**Pneum.**	normal	0.91 / 0.89	0.15 / 0.23	0.78 / 0.77	0.89 / 0.89	0.90 / 0.81	0.92 / 0.90
	pneumonia	0.91 / 0.91	0.15 / 0.44	0.78 / 0.79	0.89 / 0.90	0.90 / 0.91	0.92 / 0.94
**Retina**	0	0.76 / 0.65	0.78 / 0.66	0.79 / 0.70	0.84 / 0.83	0.81 / 0.73	0.83 / 0.78
	1	0.50 / 0.14	0.51 / 0.12	0.60 / 0.18	0.60 / 0.15	0.54 / 0.14	0.65 / 0.22
	2	0.59 / 0.30	0.65 / 0.32	0.65 / 0.35	0.68 / 0.35	0.57 / 0.34	0.70 / 0.41
	3	0.71 / 0.31	0.74 / 0.30	0.73 / 0.36	0.74 / 0.38	0.72 / 0.32	0.77 / 0.35
	4	0.65 / 0.27	0.61 / 0.16	0.64 / 0.19	0.61 / 0.07	0.63 / 0.07	0.69 / 0.16
**Breast**	malignant	0.73 / 0.50	0.58 / 0.31	0.86 / 0.66	0.82 / 0.63	0.74 / 0.49	0.75 / 0.55
	normal, benign	0.73 / 0.86	0.58 / 0.82	0.86 / 0.93	0.82 / 0.92	0.74 / 0.85	0.75 / 0.88
**Blood**	basophil	0.74 / 0.19	0.70 / 0.11	0.97 / 0.73	0.92 / 0.53	0.78 / 0.16	0.77 / 0.16
	eosinophil	0.84 / 0.59	0.61 / 0.20	0.99 / 0.97	0.98 / 0.96	0.58 / 0.27	0.99 / 0.97
	erythroblast	0.73 / 0.17	0.27 / 0.07	0.97 / 0.89	0.94 / 0.82	0.92 / 0.78	0.92 / 0.62
	immature granulocytes-, metam- and promyelocytes	0.78 / 0.44	0.75 / 0.37	0.92 / 0.66	0.90 / 0.60	0.82 / 0.46	0.88 / 0.58
	lymphocyte	0.87 / 0.51	0.33 / 0.05	0.99 / 0.88	0.96 / 0.75	0.95 / 0.50	0.90 / 0.33
	monocyte	0.87 / 0.29	0.87 / 0.31	0.93 / 0.57	0.93 / 0.51	0.65 / 0.17	0.81 / 0.23
	neutrophil	0.81 / 0.42	0.49 / 0.17	0.99 / 0.97	0.97 / 0.90	0.94 / 0.88	0.97 / 0.92
	platelet	0.99 / 0.94	1.00 / 0.98	1.00 / 1.00	1.00 / 1.00	0.99 / 0.86	1.00 / 0.99
**Tissue**	Collecting Duct, Connecting Tubule	0.57 / 0.34	0.37 / 0.25	0.81 / 0.65	0.83 / 0.70	0.77 / 0.59	0.74 / 0.55
	Distal Convoluted Tubule	0.63 / 0.07	0.55 / 0.06	0.60 / 0.06	0.65 / 0.08	0.58 / 0.06	0.58 / 0.06
	Glomerular endothelial cells	0.44 / 0.03	0.75 / 0.12	0.83 / 0.17	0.88 / 0.16	0.81 / 0.10	0.63 / 0.04
	Interstitial endothelial cells	0.60 / 0.13	0.67 / 0.16	0.86 / 0.40	0.89 / 0.45	0.80 / 0.30	0.59 / 0.12
	Leukocytes	0.48 / 0.06	0.62 / 0.11	0.64 / 0.16	0.86 / 0.30	0.74 / 0.20	0.52 / 0.07
	Podocytes	0.48 / 0.04	0.60 / 0.06	0.71 / 0.12	0.80 / 0.14	0.48 / 0.04	0.45 / 0.04
	Proximal Tubule Segments	0.74 / 0.41	0.70 / 0.38	0.81 / 0.61	0.84 / 0.65	0.76 / 0.49	0.70 / 0.39
	Thick Ascending Limb	0.59 / 0.17	0.66 / 0.23	0.72 / 0.28	0.80 / 0.40	0.71 / 0.30	0.68 / 0.27
**OrganA**	bladder	0.86 / 0.21	0.90 / 0.60	0.97 / 0.81	0.79 / 0.36	0.92 / 0.37	0.97 / 0.81
	femur-left	0.87 / 0.22	0.79 / 0.11	0.97 / 0.79	0.91 / 0.23	0.85 / 0.32	0.95 / 0.46
	femur-right	0.88 / 0.36	0.81 / 0.13	0.98 / 0.82	0.94 / 0.33	0.60 / 0.07	0.94 / 0.56
	heart	0.94 / 0.74	0.49 / 0.04	0.99 / 0.95	0.92 / 0.50	0.55 / 0.05	0.72 / 0.08
	kidney-left	0.79 / 0.28	0.59 / 0.16	0.93 / 0.68	0.81 / 0.38	0.77 / 0.32	0.85 / 0.43
	kidney-right	0.74 / 0.25	0.61 / 0.13	0.94 / 0.71	0.78 / 0.40	0.67 / 0.18	0.74 / 0.24
	liver	0.93 / 0.80	0.74 / 0.34	0.99 / 0.97	0.95 / 0.89	0.86 / 0.60	0.90 / 0.63
	lung-left	0.97 / 0.88	0.95 / 0.73	1.00 / 0.99	0.96 / 0.79	0.80 / 0.19	0.96 / 0.67
	lung-right	0.98 / 0.94	0.97 / 0.89	1.00 / 0.97	0.94 / 0.70	0.81 / 0.21	0.95 / 0.58
	pancreas	0.79 / 0.19	0.78 / 0.27	0.97 / 0.82	0.86 / 0.49	0.56 / 0.09	0.48 / 0.08
	spleen	0.72 / 0.20	0.68 / 0.20	0.92 / 0.63	0.82 / 0.39	0.71 / 0.33	0.72 / 0.29
**OrganC**	bladder	0.91 / 0.65	0.88 / 0.59	0.97 / 0.85	0.83 / 0.62	0.88 / 0.40	0.95 / 0.83
	femur-left	0.79 / 0.47	0.66 / 0.08	0.95 / 0.69	0.85 / 0.23	0.85 / 0.30	0.94 / 0.53
	femur-right	0.82 / 0.51	0.65 / 0.09	0.97 / 0.80	0.88 / 0.26	0.52 / 0.06	0.88 / 0.26
	heart	0.91 / 0.71	0.42 / 0.04	0.98 / 0.90	0.95 / 0.70	0.50 / 0.05	0.79 / 0.14
	kidney-left	0.79 / 0.30	0.63 / 0.11	0.92 / 0.63	0.83 / 0.38	0.68 / 0.21	0.85 / 0.35
	kidney-right	0.77 / 0.38	0.64 / 0.12	0.92 / 0.67	0.84 / 0.47	0.57 / 0.11	0.75 / 0.25
	liver	0.89 / 0.78	0.69 / 0.40	0.99 / 0.98	0.97 / 0.90	0.76 / 0.52	0.91 / 0.70
	lung-left	0.99 / 0.93	0.89 / 0.27	1.00 / 0.98	0.95 / 0.72	0.47 / 0.06	0.94 / 0.45
	lung-right	0.99 / 0.97	0.82 / 0.17	0.99 / 0.97	0.97 / 0.71	0.60 / 0.07	0.96 / 0.59
	pancreas	0.81 / 0.27	0.50 / 0.08	0.96 / 0.78	0.89 / 0.54	0.49 / 0.08	0.52 / 0.08
	spleen	0.72 / 0.24	0.66 / 0.16	0.95 / 0.76	0.89 / 0.54	0.69 / 0.27	0.82 / 0.57
**OrganS**	bladder	0.89 / 0.48	0.88 / 0.59	0.95 / 0.81	0.76 / 0.42	0.90 / 0.59	0.96 / 0.84
	femur-left	0.79 / 0.32	0.66 / 0.07	0.94 / 0.39	0.90 / 0.27	0.76 / 0.16	0.93 / 0.39
	femur-right	0.81 / 0.35	0.64 / 0.07	0.95 / 0.47	0.93 / 0.33	0.52 / 0.06	0.92 / 0.34
	heart	0.50 / 0.11	0.44 / 0.05	0.92 / 0.57	0.86 / 0.39	0.47 / 0.06	0.64 / 0.08
	kidney-left	0.76 / 0.24	0.56 / 0.08	0.85 / 0.36	0.80 / 0.25	0.65 / 0.14	0.79 / 0.30
	kidney-right	0.77 / 0.26	0.59 / 0.09	0.88 / 0.38	0.82 / 0.32	0.57 / 0.10	0.64 / 0.16
	liver	0.83 / 0.60	0.71 / 0.38	0.97 / 0.91	0.92 / 0.76	0.73 / 0.46	0.91 / 0.75
	lung-left	0.94 / 0.48	0.67 / 0.08	0.99 / 0.76	0.94 / 0.36	0.37 / 0.04	0.95 / 0.31
	lung-right	0.97 / 0.72	0.63 / 0.07	0.97 / 0.80	0.95 / 0.46	0.89 / 0.20	0.97 / 0.58
	pancreas	0.70 / 0.24	0.57 / 0.18	0.89 / 0.61	0.80 / 0.41	0.57 / 0.20	0.53 / 0.17
	spleen	0.65 / 0.18	0.64 / 0.15	0.81 / 0.48	0.73 / 0.32	0.62 / 0.19	0.80 / 0.41

Table S3. Per-class AUROC / AP across datasets and model types (3D datasets). AUC - Area Under the Receiver Operating characteristic, AP - Mean Average Precision. Best checkpoint selected by highest Overall Accuracy.

					SaFF-AD *(Ours)*		
Dataset	Class	**MLP** (AUROC/AP)	**CNN** (AUROC/AP)	**ResNet18** (AUROC/AP)	**FFA** (AUROC/AP)	**CFFA** (AUROC/AP)	**ResCFFA** (AUROC/AP)
**Organ**	liver	0.95 / 0.67	0.74 / 0.19	0.97 / 0.88	0.96 / 0.89	0.88 / 0.52	0.91 / 0.55
	kidney-right	0.77 / 0.46	0.45 / 0.10	0.86 / 0.49	0.75 / 0.21	0.79 / 0.42	0.74 / 0.29
	kidney-left	0.65 / 0.15	0.47 / 0.11	0.85 / 0.44	0.69 / 0.17	0.76 / 0.25	0.62 / 0.15
	femur-right	0.88 / 0.73	0.78 / 0.43	0.97 / 0.83	0.75 / 0.21	0.89 / 0.51	0.88 / 0.40
	femur-left	0.27 / 0.12	0.70 / 0.17	0.95 / 0.79	0.79 / 0.25	0.79 / 0.53	0.91 / 0.55
	bladder	0.72 / 0.17	0.76 / 0.21	0.91 / 0.73	0.84 / 0.40	0.78 / 0.50	0.81 / 0.27
	heart	0.99 / 0.92	0.45 / 0.04	0.94 / 0.79	0.59 / 0.08	0.72 / 0.16	0.53 / 0.04
	lung-right	1.00 / 1.00	0.79 / 0.12	0.94 / 0.74	0.95 / 0.28	0.85 / 0.17	0.78 / 0.07
	lung-left	0.98 / 0.82	0.77 / 0.08	0.96 / 0.78	0.96 / 0.63	0.87 / 0.16	0.82 / 0.09
	spleen	0.86 / 0.57	0.47 / 0.11	0.89 / 0.51	0.79 / 0.43	0.70 / 0.22	0.76 / 0.23
	pancreas	0.82 / 0.29	0.67 / 0.16	0.96 / 0.80	0.77 / 0.25	0.89 / 0.57	0.72 / 0.21
**Nodule**	benign	0.62 / 0.87	0.40 / 0.76	0.66 / 0.85	0.77 / 0.92	0.72 / 0.88	0.61 / 0.86
	malignant	0.62 / 0.31	0.40 / 0.16	0.66 / 0.43	0.77 / 0.42	0.71 / 0.35	0.61 / 0.31
**Adrenal**	normal	0.67 / 0.87	0.47 / 0.74	0.52 / 0.77	0.66 / 0.87	0.56 / 0.79	0.65 / 0.85
	hyperplasia	0.67 / 0.35	0.47 / 0.23	0.52 / 0.25	0.66 / 0.36	0.58 / 0.33	0.65 / 0.36
**Fracture**	buckle rib fracture	0.69 / 0.60	0.64 / 0.52	0.54 / 0.41	0.60 / 0.45	0.50 / 0.38	0.65 / 0.53
	nondisplaced rib fracture	0.59 / 0.48	0.60 / 0.51	0.56 / 0.48	0.53 / 0.50	0.50 / 0.43	0.58 / 0.49
	displaced rib fracture	0.59 / 0.24	0.54 / 0.21	0.50 / 0.18	0.54 / 0.22	0.50 / 0.19	0.59 / 0.23
**Vessel**	vessel	0.45 / 0.89	0.48 / 0.87	0.52 / 0.89	0.74 / 0.95	0.52 / 0.89	0.53 / 0.89
	aneurysm	0.45 / 0.10	0.48 / 0.11	0.52 / 0.13	0.74 / 0.36	0.52 / 0.13	0.53 / 0.14
**Synapse**	inhibitory synapse	0.42 / 0.26	0.49 / 0.27	0.49 / 0.26	0.57 / 0.32	0.52 / 0.31	0.53 / 0.29
	excitatory synapse	0.42 / 0.69	0.49 / 0.73	0.49 / 0.72	0.57 / 0.77	0.52 / 0.72	0.53 / 0.74

REFERENCES

Tan, J., Hou, B., Day, T., Simpson, J., Rueckert, D., and Kainz, B. (2021). Detecting outliers with poisson image interpolation. In *Medical Image Computing and Computer Assisted Intervention–MICCAI 2021: 24th International Conference, Strasbourg, France, September 27–October 1, 2021, Proceedings, Part V 24* (Springer), 581–591
